# Bispecific 10E8.4/iMab broadly neutralizing antibody in people with or without HIV-1: a partially randomized phase 1 trial

**DOI:** 10.1038/s41591-026-04472-w

**Published:** 2026-07-07

**Authors:** Deborah A. Theodore, Bryan T. Mayer, Charlotte-Paige Rolle, Edwin DeJesus, Margaret E. Ackerman, Michael S. Seaman, Joshua Alex Weiner, Hiroshi Mohri, Yaoxing Huang, Brett Gray, Jennifer Chang, Monica W. Gerber, Jian Yu, Yang Luo, Neal N. Padte, Michael T. Yin, Burc Barin, Rusty Greene, Steven Palmer, Ariana Pazmino, Jorge Benitez, Carlie Dorr, Meredith McNairy, James DiRenzo, Chanc VanWinkle Orzell, David D. Ho, Ollivier Hyrien, Magdalena E. Sobieszczyk

**Affiliations:** 1 Division of Infectious Diseases, Department of Medicine, Columbia University Vagelos College of Physicians and Surgeons, New York, NY USA; 2 Vaccine and Infectious Disease Division, Fred Hutchinson Cancer Center, Seattle, WA USA; 3 Orlando Immunology Center, Orlando, FL USA; 4 Department of Microbiology and Immunology, Geisel School of Medicine at Dartmouth, Hanover, NH USA; 5 Thayer School of Engineering, Dartmouth College, Hanover, NH USA; 6 Center for Virology and Vaccine Research, Beth Israel Deaconess Medical Center, Harvard Medical School, Boston, MA USA; 7 Aaron Diamond AIDS Research Center, Columbia University Vagelos College of Physicians and Surgeons, New York, NY USA; 8 The Emmes Company, LLC, Rockville, MD USA; 9 Department of Molecular and Systems Biology, Geisel School of Medicine at Dartmouth, Hanover, NH USA

**Keywords:** HIV infections, Antibody therapy, HIV infections

## Abstract

Broadly neutralizing antibodies (bnAbs) are a promising tool for HIV prevention and treatment. Here we conducted a first-in-human, phase 1 trial of the bispecific 10E8.4/iMab antibody, which consists of a 10E8.4 arm binding the HIV-1 envelope glycoprotein membrane-proximal external region and an ibalizumab (iMab) arm binding the human CD4 molecule. 10E8.4/iMab was administered intravenously (IV) or subcutaneously (SC). Safety/tolerability within 2 weeks of 10E8.4/iMab administration (primary outcome) and the pharmacokinetics (PK), antiviral activity, induction of anti-10E8.4/iMab antibodies, longitudinal CD4^+^ and CD8^+^ T cell counts and long-term safety (secondary outcomes) were evaluated. 54 participants living with HIV (PLWH) or without HIV (PLWoH) received 10E8.4/iMab or placebo. In arm 1, PLWoH received 10E8.4/iMab 0.3 mg kg^−1^ IV, 1 mg kg^−1^ SC, or 1 mg kg^−1^ IV (*n* = 3 each). In arm 2, PLWoH received 10E8.4/iMab 3 mg kg^−1^ IV, 10 mg kg^−1^ IV or 30 mg kg^−1^ IV (*n* = 6 each). In arms 3/3a, PLWH received 10E8.4/iMab 10 mg kg^−1^ IV (*n* = 3) or 30 mg kg^−1^ IV (*n* = 6). In arm 4, PLWoH were randomized to receive 10E8.4/iMab or placebo 2.5 mg kg^−1^ SC or 10 mg kg^−1^ SC (*n* = 9 each). Participants in arms 1–3 were not randomized. No treatment-related serious adverse events (AEs) or AEs ≥ grade 3 were reported. The most common solicited AEs were tenderness (10/54, 18.5%), fatigue (18/54, 33.3%) and headache (12/54, 22.2%). Related grade 2 local and systemic solicited AEs occurred in one and six participants, respectively. Three of nine PLWH developed a generalized rash 8–12 days after infusion that resolved within 9–16 days. The primary objective of the study to evaluate the safety/tolerability of 10E8.4/iMab was met. These data support further study of 10E8.4/iMab to expand HIV treatment and prevention options. ClinicalTrials.gov: NCT03875209.

## Abstract (web summary)

A first-in-human, partially randomized and placebo-controlled phase I trial examined the safety, tolerability, pharmacokinetics and antiviral activity of the bispecific 10E8.4/iMab broadly neutralizing monoclonal antibody in people living with or without HIV, and found it was safe and well tolerated at different dose levels and routes of administration.

## Main

Despite significant advances in human immunodeficiency virus (HIV) treatment and prevention over the past four decades, approximately 40.8 million people are living with HIV, and there are approximately 1.3 million new infections per year^1^. Given recent disruptions to global public health infrastructure, these numbers may increase^2^. Long-acting formulations of highly effective antiretrovirals for HIV treatment and prevention have become available in some settings^3^, but barriers to access persist, and preferences for long-acting products differ according to the population surveyed^4^. To end the HIV epidemic, it will be essential to expand the portfolio of long-acting options that are safe, tolerable and scalable so that people who stand to benefit can choose the option that works best for them^5^.

Broadly neutralizing antibodies (bnAbs) are a promising tool to control HIV viremia, reduce the size of the viral reservoir^6^, and prevent HIV acquisition^7,8^. Realizing the full potential of bnAbs will likely require combination therapy (for example, using multiple bnAbs that bind different targets) or bi- or tri-specific bnAb therapy (single molecules that bind multiple targets)^9–11^.

**Extended Data Fig. 1:**
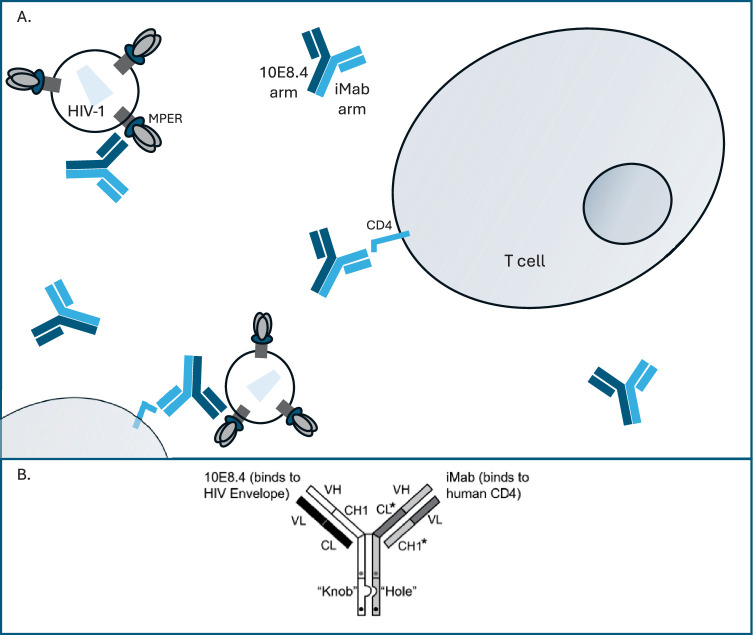
10E8.4/iMab Schematic. **a**. 10E8.4/iMab consists of one arm of ibalizumab (iMab, light blue), a humanized monoclonal antibody that binds human CD4, and one arm of 10E8.4 (dark blue), a monoclonal antibody that binds the HIV-1 glycoprotein 41 (gp41) membrane-proximal external region (MPER), which is a highly conserved site on the viral envelope. Not to scale. **b**. The creation of a ‘knob’ in one heavy chain and a ‘hole’ in the other heavy chain supports the formation of heavy chain heterodimers, while the ‘crossover’ of CL and CH1 sequences in one arm of the antibody supports correct heavy-light chain pairings in both arms^18^.

Immunotherapy with bispecific bnAbs is increasingly studied for a range of viral infections, as well as for oncologic and other non-oncologic indications^12–16^. The bispecific bnAb 10E8.4/iMab, one of the most potent and broad HIV bnAbs developed to date, neutralizes nearly all circulating HIV-1 strains tested and showed efficacy in preventing and treating HIV-1 in a humanized mouse model^17,18^. 10E8.4/iMab consists of one arm of 10E8.4, a monoclonal antibody that binds the HIV-1 glycoprotein 41 membrane-proximal external region, which is a highly conserved site on the viral envelope important for viral fusion (Extended Data Fig. 1)^19^, and one arm of ibalizumab (iMab), a humanized monoclonal antibody that binds human CD4 and thereby blocks HIV entry^20^. The linkage of these arms leads to marked synergistic enhancement of neutralization breadth and potency^17^. 10E8.4/iMab was additionally engineered to have an enhanced in vivo half-life and reduced Fc-effector functions, such as antibody-dependent cell-mediated cytotoxicity and complement-dependent cytotoxicity, to decrease potential IgG-mediated toxicity; iMab itself was developed as an IgG4 subclass monoclonal antibody to minimize Fc-effector functions^21–24^.

We conducted a phase 1 first-in-human trial to evaluate the safety, tolerability, pharmacokinetics (PK) and antiviral activities of the bispecific 10E8.4/iMab antibody given intravenously in people living with HIV (PLWH) and people living without HIV (PLWoH) and given subcutaneously in PLWoH.

## Results

### Trial design and participant disposition

Sixty-three participants were screened to determine eligibility. Eligible participants were 18–60 years of age and in good health. Eligibility for specific study arms and groups varied by HIV status and HIV-1 RNA level. PLWH were eligible for arm 3 group H (10 mg kg^−1^ IV) participation if their CD4^+^ T cell count was >350 cells mm^−^^3^ and HIV-1 RNA was between 1,000 and 100,000 copies ml^−1^ in the context of being treatment-naïve, off treatment for at least 4 weeks in consultation with their primary provider or clinically stable and without recent changes to their treatment regimen. PLWH were eligible for arm 3a group I (30 mg kg^−1^ IV) participation if their CD4^+^ T cell count was >350 cells mm^−^^3^ and HIV-1 RNA was undetectable on triple-combination antiretroviral therapy.

**Fig. 1:**
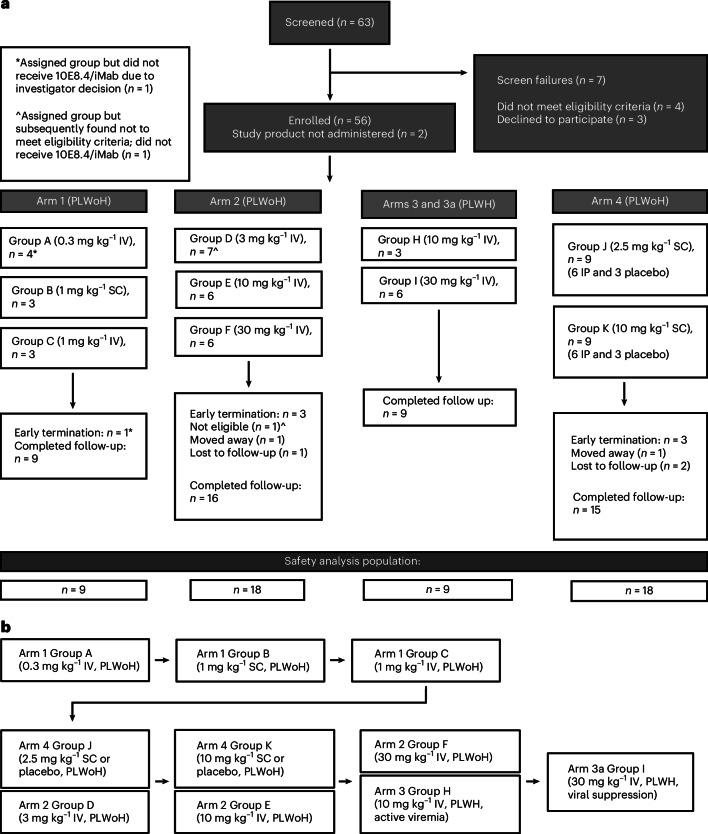
Trial profile. **a**, Consort diagram. **b**, Sequence of arm and group enrollments. Arm 1 group A enrolled first, followed by arm 1 group B and then arm 1 group C. Thereafter, arm 4 group J and arm 2 group D enrolled contemporaneously; if a participant was eligible for both groups, and if a group assignment was open for both groups, then the participant could choose whether to enter arm 4 group J (and thereafter undergo randomization to 10E8.4/iMab or placebo) or arm 2 group D. Next, arm 4 group K and arm 2 group E enrolled contemporaneously, with the same group assignment process as arm 4 group J/arm 2 group D. Next, arm 2 group F and arm 3 group H enrolled contemporaneously, with group assignment determined by HIV status. Finally, arm 3a group I enrolled.

Fifty-four participants enrolled and received 10E8.4/iMab or placebo between 21 March 2019 and 1 October 2021. Arm 1 group A (0.3 mg kg^−1^ IV, PLWoH; *n* = 3) participants enrolled first. Arm 1 group B (1 mg kg^−1^ SC, PLWoH; *n* = 3) enrolled next. Arm 1 group C (1 mg kg^−1^ IV, PLWoH; *n* = 3) enrolled next. Thereafter, participants in arm 4 group J (2.5 mg kg^−1^ SC or placebo, PLWoH; *n* = 9) and arm 2 group D (3 mg kg^−1^ IV, PLWoH; *n* = 6) enrolled contemporaneously; if a participant was eligible for both groups and a group assignment was open for both, the participant could choose whether to enter arm 4 group J (and thereafter undergo randomization to 10E8.4/iMab or placebo) or arm 2 group D. Next, participants in arm 4 group K (10 mg kg^−1^ SC or placebo, PLWoH; *n* = 9) and arm 2 group E (10 mg kg^−1^ IV, PLWoH; *n* = 6) enrolled contemporaneously; similarly, if a participant was eligible for both groups and a group assignment was open for both, the participant could choose whether to enter arm 4 group K (and thereafter undergo randomization to 10E8.4/iMab or placebo) or arm 2 group E. Next, participants in arm 2 group F (30 mg kg^−1^ IV, PLWoH; *n* = 6) and arm 3 group H (10 mg kg^−1^ IV, PLWH with active viremia; *n* = 3) enrolled contemporaneously, with group assignment determined by HIV status. Finally, participants in arm 3a group I (30 mg kg^−1^ IV, PLWH with viral suppression; *n* = 6) enrolled (Fig. 1).

There were eight protocol amendments (described in Methods). The study opened under Protocol Amendment 2. Fifty-three protocol deviations were reported under the following categories: protocol procedure/assessment (33/53, 62.3%), follow-up visit schedule (14/53, 26.4%), eligibility/enrollment (3/53, 5.7%), Standard Operating Procedures for Good Clinical Practice guidelines (2/53, 3.8%) and investigational product administration/dosing (1/53, 1.9%). Among the eligibility/enrollment deviations, two participants had at least one screening lab that was outside the allowable screening window at enrollment, and one participant had an exclusionary calcium level at enrollment. The investigational product administration/dosing deviation was related to priming of 10E8.4/iMab with incorrect IV line tubing; however, the deviation was noted before the infusion began, and new product was obtained and administered to the participant via the correct tubing.

Forty-nine participants completed the study; five participants left early due to moving away (*n* = 2) or loss to follow-up (*n* = 3). One follow-up visit was missed, and two follow-up visits were performed out of window due to COVID-19-like symptoms or COVID-19 diagnosis.

Median age was 27.5 years, and 26 (48.1%) were assigned female at birth. Sex was not evenly distributed between groups; arms 1, 2 and 4, which enrolled PLWoH, included 50–67% female participants, whereas arms 3 and 3a, which enrolled PLWH, included no female participants. Half of participants identified as White (27/54, 50.0%), followed by Black or African American (9/54, 16.7%) and other/unknown/refused to specify (9/54, 16.7%), Asian (8/54, 14.8%) and multiracial (1/54, 1.8%). Twenty-one participants (38.9%) reported Latino or Hispanic ethnicity (Table 1). Among PLWH, median years from diagnosis was 2.9 (range 0.1–29.0), and nearly all antiretroviral treatment (ART) regimens included an integrase strand transfer inhibitor, except for one regimen that combined two nucleoside reverse transcriptase inhibitors with one non-nucleoside reverse transcriptase inhibitor. At enrollment, group H (viremic) participants agreed (in consultation with their primary provider) not to begin or change ART for 6 weeks after 10E8.4/iMab infusion, despite a clear explanation of HIV treatment guidelines. Study investigators discussed each PLWH with the participant’s HIV primary provider, with the understanding that ART could be started, restarted, or switched at any time if clinically indicated. All group H participants were on effective ART with HIV VL <50 copies ml^−1^ at end of study.

**Table 1:** Demographic characteristics of study participants (safety population) at baseline

	arm 1 (*n* = 9)	arm 2 (*n* = 18)	arm 3/3a (*n* = 9)	arm 4 (*n* = 18)
**Sex at birth,** ***n*** **(%)**				
Female	6 (67)	9 (50)	0 (0)	11 (61)
Male	3 (33)	9 (50)	9 (100)	7 (39)
**Gender,** ***n*** **(%)**				
Female	6 (67)	9 (50)	0 (0)	11 (61)
Male	3 (33)	9 (50)	9 (100)	7 (39)
Other	0 (0)	0 (0)	0 (0)	0 (0)
**Age (y), median (range)**				
	28 (21–37)	33.5 (19–55)	47 (28–58)	25 (18–37)
**Weight (kg), median (range)**				
	75 (47–96)	82 (55–99)	71 (55–107)	67 (49–75)
**Ethnicity,** ***n*** **(%)**				
Hispanic or Latino	3 (33)	9 (50)	4 (44)	5 (28)
Not Hispanic or Latino	6 (67)	9 (50)	5 (56)	13 (72)
**Race,** ***n*** **(%)**				
Asian	2 (22)	0 (0)	1 (11)	6 (33)
Black	1 (11)	3 (17)	3 (33)	2 (11)
White	5 (56)	14 (78)	2 (22)	6 (33)
Other	1 (11)	1 (6)	3 (33)	4 (22)

The primary outcome was the rate of signs, symptoms and laboratory abnormalities, in addition to local and systemic solicited adverse events (AEs), within 2 weeks of 10E8.4/iMab administration in all study arms/groups. Secondary outcomes were the PK profile of 10E8.4/iMab (elimination half-life (t_1/2_), clearance (CL/F), volume of distribution (Vz/F), area under the concentration-time curve (AUC) and decay curve in all study arms/groups); the decline in plasma HIV-1 RNA level by standard clinical assay after 10E8.4/iMab infusion in PLWH with viremia; the frequency and levels of induced anti-10E8.4/iMab antibodies in all study groups; the rate of signs, symptoms and laboratory abnormalities that occurred during study follow-up after 10E8.4/iMab infusion/injection in all study groups; and the absolute and relative CD4^+^ and CD8^+^ T cell counts after 10E8.4/iMab infusion. We reported the noncompartmental PK analysis by group for drug exposure (AUC), but because of the non-linear PK for 10E8.4/iMab, the remaining PK outcomes were reported using population modeling across groups by cohort, separated by PLWH versus PLWoH. All outcomes are presented here.

### Primary outcome

Among PLWoH, grade 1 or higher AEs (solicited or unsolicited signs, symptoms and laboratory abnormalities, related or unrelated) occurred within 2 weeks of 10E8.4/iMab administration at the following frequencies: 66.7% (2/3) among group A (0.3 mg kg^−1^ IV), 66.7% (2/3) among group B (1 mg kg^−1^ SC), 100% (3/3) among group C (1 mg kg^−1^ IV), 66.7% (4/6) among group D (3 mg kg^−1^ IV), 83.3% (5/6) among group E (10 mg kg^−1^ IV), 100% (6/6) among group F (30 mg kg^−1^ IV), 66.7% (4/6) among group J (2.5 mg kg^−1^ SC) and 100% (6/6) among group K (10 mg kg^−1^ SC). Among placebo recipients, 83.3% (5/6) reported a grade 1 or higher AE. Among PLWH, 66.7% (2/3) of participants in group H (10 mg kg^−1^ IV) and 83.3% (5/6) of participants in group I (30 mg kg^−1^ IV) reported a grade 1 or higher AE (Table 2).

**Table 2:** Solicited AEs and unsolicited AEs (signs, symptoms and laboratory abnormalities) occurring within 2 weeks of 10E8.4/iMab administration in all study arms/groups

	Arm 1 (PLWoH)			Arm 2 (PLWoH)			Arm 3/3a (PLWH)		Arm 4 (PLWoH)		
	Group A	Group B	Group C	Group D	Group E	Group F	Group H	Group I	Group J	Group K	Groups J-K
	0.3 mg kg^−1^ IV (*n* = 3)	1 mg kg^−1^ SC (*n* = 3)	1 mg kg^−1^ IV (*n* = 3)	3 mg kg^−1^ IV (*n* = 6)	10 mg kg^−1^ IV (*n* = 6)	30 mg kg^−1^ IV (*n* = 6)	10 mg kg^−1^ IV (*n* = 3)	30 mg kg^−1^ IV (*n* = 6)	2.5 mg kg^−1^ SC (*n* = 6)	10 mg kg^−1^ SC (*n* = 6)	Placebo SC (*n* = 6)
**Any AE**	2 (66.7)	2 (66.7)	3 (100.0)	4 (66.7)	5 (83.3)	6 (100.0)	2 (66.7)	5 (83.3)	4 (66.7)	6 (100.0)	5 (83.3)
	(9.4, 99.2)	(9.4, 99.2)	(29.2, 100)	(22.3, 95.7)	(35.9, 99.6)	(54.1, 100)	(9.4, 99.2)	(35.9, 99.6)	(22.3, 95.7)	(54.1, 100)	(35.9, 99.6)
Related	1 (33.3)	2 (66.7)	1 (33.3)	3 (50.0)	4 (66.7)	4 (66.7)	2 (66.7)	5 (83.3)	2 (33.3)	6 (100.0)	3 (50.0)
	(0.8, 90.6)	(9.4, 99.2)	(0.8, 90.6)	(11.8, 88.2)	(22.3, 95.7)	(22.3, 95.7)	(9.4, 99.2)	(35.9, 99.6)	(4.3, 77.7)	(54.1, 100)	(11.8, 88.2)
Grade ≥2	1 (33.3)	0 (0.0)	2 (66.7)	1 (16.7)	2 (33.3)	4 (66.7)	2 (66.7)	4 (66.7)	2 (33.3)	1 (16.7)	2 (33.3)
	(0.8, 90.6)	(0.0, 70.8)	(9.4, 99.2)	(0.4, 64.1)	(4.3, 77.7)	(22.3, 95.7)	(9.4, 99.2)	(22.3, 95.7)	(4.3, 77.7)	(0.4, 64.1)	(4.3, 77.7)
Related and grade 2	0 (0.0)	0 (0.0)	0 (0.0)	0 (0.0)	1 (16.7)	0 (0.0)	2 (66.7)	3 (50.0)	0 (0.0)	1 (16.7)	0 (0.0)
	(0.0, 70.8)	(0.0, 70.8)	(0.0, 70.8)	(0.0, 45.9)	(0.4, 64.1)	(0.0, 45.9)	(9.4, 99.2)	(11.8, 88.2)	(0.0, 45.9)	(0.4, 64.1)	(0.0, 45.9)
Related and grade 3 or 4	0 (0.0)	0 (0.0)	0 (0.0)	0 (0.0)	0 (0.0)	0 (0.0)	0 (0.0)	0 (0.0)	0 (0.0)	0 (0.0)	0 (0.0)
	(0.0, 70.8)	(0.0, 70.8)	(0.0, 70.8)	(0.0, 45.9)	(0.0, 45.9)	(0.0, 45.9)	(0.0, 70.8)	(0.0, 45.9)	(0.0, 45.9)	(0.0, 45.9)	(0.0, 45.9)
**Any solicited symptom**	2 (66.7)	2 (66.7)	1 (33.3)	3 (50.0)	4 (66.7)	5 (83.3)	2 (66.7)	5 (83.3)	4 (66.7)	6 (100.0)	5 (83.3)
	(9.4, 99.2)	(9.4, 99.2)	(0.8, 90.6)	(11.8, 88.2)	(22.3, 95.7)	(35.9, 99.6)	(9.4, 99.2)	(35.9, 99.6)	(22.3, 95.7)	(54.1, 100)	(35.9, 99.6)
Local symptom	1 (33.3)	2 (66.7)	1 (33.3)	1 (16.7)	2 (33.3)	1 (16.7)	1 (33.3)	3 (50.0)	2 (33.3)	5 (83.3)	2 (33.3)
	(0.8, 90.6)	(9.4, 99.2)	(0.8, 90.6)	(0.4, 64.1)	(4.3, 77.7)	(0.4, 64.1)	(0.8, 90.6)	(11.8, 88.2)	(4.3, 77.7)	(35.9, 99.6)	(4.3, 77.7)
Related	1 (33.3)	2 (66.7)	1 (33.3)	1 (16.7)	2 (33.3)	1 (16.7)	0 (0.0)	3 (50.0)	2 (33.3)	5 (83.3)	2 (33.3)
	(0.8, 90.6)	(9.4, 99.2)	(0.8, 90.6)	(0.4, 64.1)	(4.3, 77.7)	(0.4, 64.1)	(0.0, 70.8)	(11.8, 88.2)	(4.3, 77.7)	(35.9, 99.6)	(4.3, 77.7)
Grade ≥2	0 (0.0)	0 (0.0)	0 (0.0)	0 (0.0)	0 (0.0)	0 (0.0)	0 (0.0)	0 (0.0)	0 (0.0)	1 (16.7)	0 (0.0)
	(0.0, 70.8)	(0.0, 70.8)	(0.0, 70.8)	(0.0, 45.9)	(0.0, 45.9)	(0.0, 45.9)	(0.0, 70.8)	(0.0, 45.9)	(0.0, 45.9)	(0.4, 64.1)	(0.0, 45.9)
Related and grade 2	0 (0.0)	0 (0.0)	0 (0.0)	0 (0.0)	0 (0.0)	0 (0.0)	0 (0.0)	0 (0.0)	0 (0.0)	1 (16.7)	0 (0.0)
	(0.0, 70.8)	(0.0, 70.8)	(0.0, 70.8)	(0.0, 45.9)	(0.0, 45.9)	(0.0, 45.9)	(0.0, 70.8)	(0.0, 45.9)	(0.0, 45.9)	(0.4, 64.1)	(0.0, 45.9)
Related and grade 3 or 4	0 (0.0)	0 (0.0)	0 (0.0)	0 (0.0)	0 (0.0)	0 (0.0)	0 (0.0)	0 (0.0)	0 (0.0)	0 (0.0)	0 (0.0)
	(0.0, 70.8)	(0.0, 70.8)	(0.0, 70.8)	(0.0, 45.9)	(0.0, 45.9)	(0.0, 45.9)	(0.0, 70.8)	(0.0, 45.9)	(0.0, 45.9)	(0.0, 45.9)	(0.0, 45.9)
Systemic symptom	2 (66.7)	2 (66.7)	0 (0.0)	3 (50.0)	4 (66.7)	5 (83.3)	2 (66.7)	5 (83.3)	4 (66.7)	2 (33.3)	4 (66.7)
	(9.4, 99.2)	(9.4, 99.2)	(0.0, 70.8)	(11.8, 88.2)	(22.3, 95.7)	(35.9, 99.6)	(9.4, 99.2)	(35.9, 99.6)	(22.3, 95.7)	(4.3, 77.7)	(22.3, 95.7)
Related	0 (0.0)	2 (66.7)	0 (0.0)	2 (33.3)	4 (66.7)	3 (50.0)	2 (66.7)	4 (66.7)	0 (0.0)	2 (33.3)	1 (16.7)
	(0.0, 70.8)	(9.4, 99.2)	(0.0, 70.8)	(4.3, 77.7)	(22.3, 95.7)	(11.8, 88.2)	(9.4, 99.2)	(22.3, 95.7)	(0.0, 45.9)	(4.3, 77.7)	(0.4, 64.1)
Grade ≥2	0 (0.0)	0 (0.0)	0 (0.0)	0 (0.0)	1 (16.7)	0 (0.0)	2 (66.7)	4 (66.7)	0 (0.0)	0 (0.0)	2 (33.3)
	(0.0, 70.8)	(0.0, 70.8)	(0.0, 70.8)	(0.0, 45.9)	(0.4, 64.1)	(0.0, 45.9)	(9.4, 99.2)	(22.3, 95.7)	(0.0, 45.9)	(0.0, 45.9)	(4.3, 77.7)
Related and grade 2	0 (0.0)	0 (0.0)	0 (0.0)	0 (0.0)	1 (16.7)	0 (0.0)	2 (66.7)	3 (50.0)	0 (0.0)	0 (0.0)	0 (0.0)
	(0.0, 70.8)	(0.0, 70.8)	(0.0, 70.8)	(0.0, 45.9)	(0.4, 64.1)	(0.0, 45.9)	(9.4, 99.2)	(11.8, 88.2)	(0.0, 45.9)	(0.0, 45.9)	(0.0, 45.9)
Related and grade 3 or 4	0 (0.0)	0 (0.0)	0 (0.0)	0 (0.0)	0 (0.0)	0 (0.0)	0 (0.0)	0 (0.0)	0 (0.0)	0 (0.0)	0 (0.0)
	(0.0, 70.8)	(0.0, 70.8)	(0.0, 70.8)	(0.0, 45.9)	(0.0, 45.9)	(0.0, 45.9)	(0.0, 70.8)	(0.0, 45.9)	(0.0, 45.9)	(0.0, 45.9)	(0.0, 45.9)
Infusion reaction	0 (0.0)	0 (0.0)	0 (0.0)	0 (0.0)	0 (0.0)	0 (0.0)	0 (0.0)	0 (0.0)	0 (0.0)	0 (0.0)	0 (0.0)
	(0.0, 70.8)	(0.0, 70.8)	(0.0, 70.8)	(0.0, 45.9)	(0.0, 45.9)	(0.0, 45.9)	(0.0, 70.8)	(0.0, 45.9)	(0.0, 45.9)	(0.0, 45.9)	(0.0, 45.9)
**Any unsolicited AE**	2 (66.7)	0 (0.0)	2 (66.7)	2 (33.3)	3 (50.0)	6 (100.0)	1 (33.3)	3 (50.0)	3 (50.0)	0 (0.0)	2 (33.3)
	(9.4, 99.2)	(0.0, 70.8)	(9.4, 99.2)	(4.3, 77.7)	(11.8, 88.2)	(54.1, 100)	(0.8, 90.6)	(11.8, 88.2)	(11.8, 88.2)	(0.0, 45.9)	(4.3, 77.7)
Related	0 (0.0)	0 (0.0)	0 (0.0)	0 (0.0)	0 (0.0)	2 (33.3)	1 (33.3)	0 (0.0)	0 (0.0)	0 (0.0)	0 (0.0)
	(0.0, 70.8)	(0.0, 70.8)	(0.0, 70.8)	(0.0, 45.9)	(0.0, 45.9)	(4.3, 77.7)	(0.8, 90.6)	(0.0, 45.9)	(0.0, 45.9)	(0.0, 45.9)	(0.0, 45.9)
Grade ≥2	1 (33.3)	0 (0.0)	2 (66.7)	1 (16.7)	2 (33.3)	4 (66.7)	0 (0.0)	1 (16.7)	2 (33.3)	0 (0.0)	1 (16.7)
	(0.8, 90.6)	(0.0, 70.8)	(9.4, 99.2)	(0.4, 64.1)	(4.3, 77.7)	(22.3, 95.7)	(0.0, 70.8)	(0.4, 64.1)	(4.3, 77.7)	(0.0, 45.9)	(0.4, 64.1)
Related and grade 2	0 (0.0)	0 (0.0)	0 (0.0)	0 (0.0)	0 (0.0)	0 (0.0)	0 (0.0)	0 (0.0)	0 (0.0)	0 (0.0)	0 (0.0)
	(0.0, 70.8)	(0.0, 70.8)	(0.0, 70.8)	(0.0, 45.9)	(0.0, 45.9)	(0.0, 45.9)	(0.0, 70.8)	(0.0, 45.9)	(0.0, 45.9)	(0.0, 45.9)	(0.0, 45.9)
Related and grade 3 or 4	0 (0.0)	0 (0.0)	0 (0.0)	0 (0.0)	0 (0.0)	0 (0.0)	0 (0.0)	0 (0.0)	0 (0.0)	0 (0.0)	0 (0.0)
	(0.0, 70.8)	(0.0, 70.8)	(0.0, 70.8)	(0.0, 45.9)	(0.0, 45.9)	(0.0, 45.9)	(0.0, 70.8)	(0.0, 45.9)	(0.0, 45.9)	(0.0, 45.9)	(0.0, 45.9)
**Any serious AE**	0 (0.0)	0 (0.0)	0 (0.0)	0 (0.0)	0 (0.0)	0 (0.0)	0 (0.0)	0 (0.0)	0 (0.0)	0 (0.0)	0 (0.0)
	(0.0, 70.8)	(0.0, 70.8)	(0.0, 70.8)	(0.0, 45.9)	(0.0, 45.9)	(0.0, 45.9)	(0.0, 70.8)	(0.0, 45.9)	(0.0, 45.9)	(0.0, 45.9)	(0.0, 45.9)
Any grade ≥1 laboratory result	2 (66.7) (9.4, 99.2)	2 (66.7) (9.4, 99.2)	3 (100.0) (29.2, 100)	6 (100.0) (54.1, 100)	4 (66.7) (22.3, 95.7)	6 (100.0) (54.1, 100)	2 (66.7) (9.4, 99.2)	4 (66.7) (22.3, 95.7)	4 (66.7) (22.3, 95.7)	4 (66.7) (22.3, 95.7)	5 (83.3) (35.9, 99.6)
Any grade ≥2 laboratory result	1 (33.3) (0.8, 90.6)	0 (0.0) (0.0, 70.8)	2 (66.7) (9.4, 99.2)	1 (16.7) (0.4, 64.1)	1 (16.7) (0.4, 64.1)	2 (33.3) (4.3, 77.7)	0 (0.0) (0.0, 70.8)	1 (16.7) (0.4, 64.1)	2 (33.3) (4.3, 77.7)	1 (16.7) (0.4, 64.1)	1 (16.7) (0.4, 64.1)

Values are reported as *n* (%) (95% CI).

Among PLWoH, grade 1 or higher related solicited AEs (local or systemic reactogenicity) occurred within 2 weeks of 10E8.4/iMab administration at the following frequencies: 33.3% (1/3) among group A, 66.7% (2/3) among group B, 33.3% (1/3) among group C, 50.0% (3/6) among group D, 66.7% (4/6) among group E, 66.7% (4/6) among group F, 33.3% (2/6) among group J and 100.0% (6/6) among group K. Among placebo recipients, 50% (3/6) reported a grade 1 or higher related solicited AE. Among PLWH, 66.7% (2/3) of participants in group H and 83.3% (5/6) of participants in group I reported a grade 1 or higher related solicited AE (Extended Data Table 1).

The most common local solicited AE (related or unrelated) was tenderness, which was reported by 18.5% (10/54) of study participants. The most common systemic solicited AEs (related or unrelated) were fatigue (33.3%, 18/54) and headache (22.2%, 12/54). All related solicited AEs were classified as grade 1 (mild) or grade 2 (moderate). Only one participant (group K, 10 mg kg^−1^ SC, randomized to 10E8.4/iMab) reported related grade 2 local symptoms (swelling, warmth, itching and erythema). Six participants reported related grade 2 systemic symptoms; of these, five were PLWH, including three PLWH who developed a generalized and occasionally pruritic grade 2 rash between days 8 and 12 following infusion. This rash developed in two of three individuals in group H and one of six participants in group I. One group H participant developed grade 2 headache, fever, fatigue and pruritus along with the rash. All rashes resolved with conservative medical management (range 9–16 days until resolution) (Extended Data Table 1). Additionally, three unrelated grade 3 (severe) solicited AEs (headache, myalgia and fatigue) occurred in a placebo recipient with influenza.

There were no acute infusion reactions following 10E8.4/iMab administration. No deaths, serious AEs, pregnancies or dose-limiting AEs were reported within 2 weeks of enrollment.

### Secondary outcomes

#### Long-term safety

Among PLWoH, grade 1 or higher AEs (solicited or unsolicited signs, symptoms and laboratory abnormalities, related or unrelated) occurred during study follow-up (after 10E8.4/iMab administration) at the following frequencies: 66.7% (2/3) among group A, 100% (3/3) among group B, 100% (3/3) among group C, 83.3% (5/6) among group D, 100% (6/6) among group E, 100% (6/6) among group F, 66.7% (4/6) among group J and 100% (6/6) among group K. Among placebo recipients, 83.3% (5/6) reported a grade 1 or higher AE. Among PLWH, 100% (3/3) of participants in group H (10 mg kg^−1^ IV) and 100% (6/6) of participants in group I (30 mg kg^−1^ IV) reported a grade 1 or higher AE (Extended Data Table 2).

In total, 90 unsolicited AEs were reported by 37/54 (68.5%) participants over the study. No deaths, serious AEs, pregnancies or dose-limiting AEs were reported. One grade 4 (potentially life threatening) hypoglycemia AE was reported in a group C participant with a glucose level of 37 mg dl^−1^ on day 0; the blood sample was drawn before 10E8.4/iMab administration and resulted after the visit. The participant had not eaten before the visit but ate during the visit and was asymptomatic; their follow-up glucose levels during the study were normal.

#### CD4^+^ and CD8^+^ T cell counts and percentages over time

**Extended Data Fig. 2:**
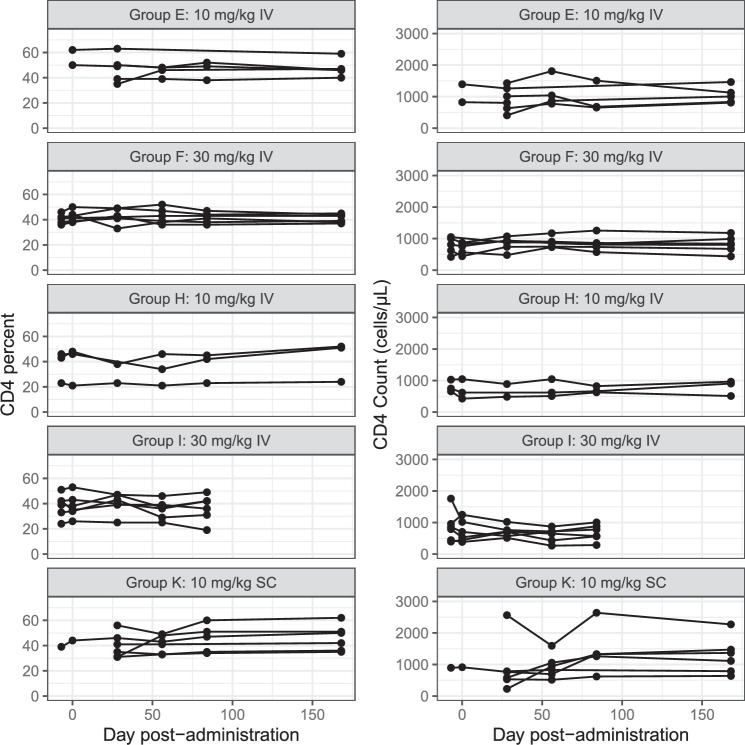
CD4+ T cell counts and percentages. CD4+ cell counts and CD4+ cells as a percentage of total lymphocytes over time in PLWoH (Groups E, F, and K) and PLWH (Groups H and I).

**Extended Data Fig. 3:**
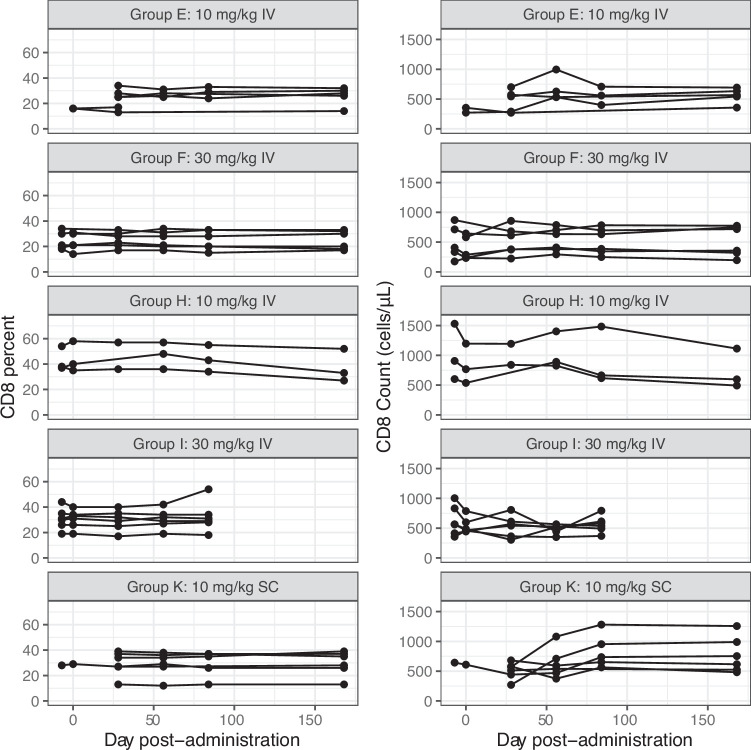
CD8+ T cell counts and percentages. CD8+ cell counts and CD8+ cells as a percentage of total lymphocytes over time in PLWoH (Groups E, F, and K) and PLWH (Groups H and I).

CD4^+^ and CD8^+^ T cell counts and percentages were measured during screening, at enrollment, and at days 28, 56, 84 and 168. T cell subsets were planned to be measured only among PLWH. After interim laboratory analysis revealed high anti-drug antibody (ADA) prevalence in arm 1 participants, the protocol was amended for additional safety monitoring to screen for potential ADA-related CD4^+^ cell count depletion; T cell subsets were thereafter measured among all participants. As a result, most T cell subset data were obtained among participants within the high-dose groups (10 mg kg^−1^ or 30 mg kg^−1^: groups E, F, H, I and K), which enrolled later. CD4^+^ and CD8^+^ counts and percentages remained stable in both cohorts (Extended Data Figs. 2 and 3); total lymphocyte counts remained stable as well (Supplementary Fig. 1).

#### PK in PLWoH

**Fig. 2:**
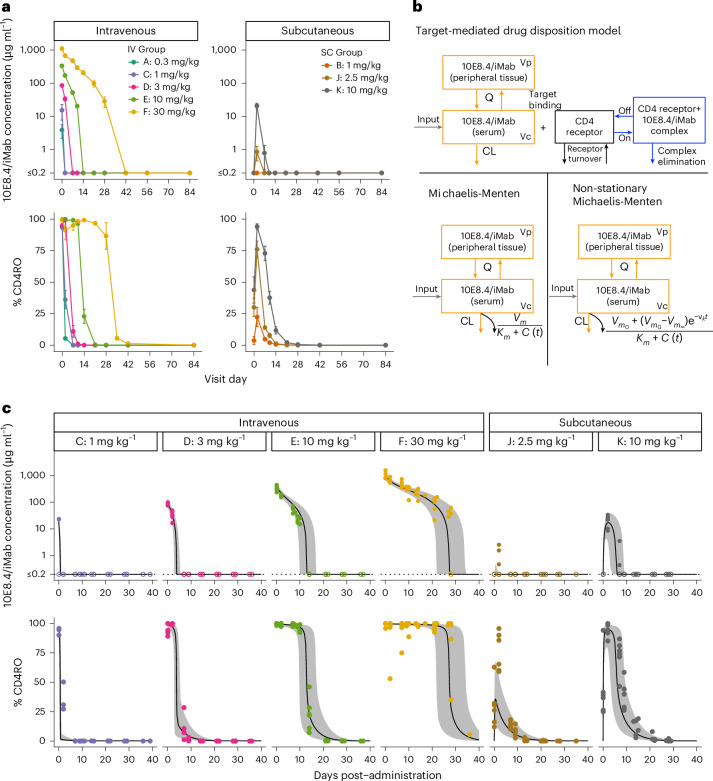
10E8.4/iMab serum concentration and percent CD4 receptor (CD4RO) levels by dose and route group (colors) among participants living without HIV (PLWoH). **a**, Mean (lines) and standard errors (error bars) of observed serum concentrations over time by group (groups A–C, *n* = 3; all other groups, *n* = 6). The first timepoint for the IV groups corresponds to 1 h after infusion. **b**, Structural PK/PD models describing serum 10E8.4/iMab kinetics and binding dynamics to CD4 receptors. The TMDD model depicts the full framework and the Michaelis-Menten (MM) models represent a simplification of the CD4 receptor kinetics and binding via a non-linear clearance term for serum 10E8.4/iMab. Each model is described by a central volume (*V*_*c*_), clearance from the central compartment (*CL*), intercompartmental clearance (*Q*) and peripheral volume (*V*_*p*_). The non-linear clearance is described by an MM process parameterized by *V*_*m*_ (maximum binding velocity) and *K*_*m*_ (concentration of half-maximum binding velocity). The non-stationary MM model best described the PK/PD for PLWoH where CD4 receptor recycling was influenced by 10E8.4/iMab, and the *V*_*m*_ term changes from *V*_*m*0_ to *V*_*m****∞***_ via log-linear rate *v*_*k*_ (see Table 3 for fitted values). **c**, Population mean (lines) and 90% prediction interval (shaded area) based on the fitted non-stationary MM model; points represent observed data. Groups A and B not depicted due to limited observed serum concentrations after administration (no positive concentrations in group B).

10E8.4/iMab serum concentration in PLWoH receiving active product exhibited non-linear PK, as evidenced by decay curves showing accelerating elimination as concentrations decreased concurrently with rapid reduction in CD4 receptor occupancy (CD4RO) (Fig. 2a). The relationship between serum concentration and CD4RO kinetics indicated a target-mediated drug disposition (TMDD) system where drug PK is affected by host target binding (here, the iMab arm binding CD4 receptors). The final day of detectable concentration and maximal CD4RO were generally aligned. Full CD4 receptor saturation (100% measured occupancy) over multiple timepoints was only achieved in participants receiving 10 or 30 mg kg^−1^ IV (groups E and F). Notably, in group F, one participant had a relatively low CD4RO value (53%) at visit 2 and another had a CD4RO value of 75% at visit 3; although these were attributed to hemolysis, CDR4O data were analyzed as measured, inclusive of those values.

Consistent with a TMDD PK profile, drug exposure estimates for 10E8.4/iMab serum concentration PK, as measured by AUC, are dose-dependent, nonlinearly increasing with increasing dose (Supplementary Table 1).

A population compartmental PK analysis was performed to construct a model that mechanistically described the PK and pharmacodynamics (PD) of serum 10E8.4/iMab concentrations and corresponding CD4RO (Methods). Serum PK was modeled using a two-compartment model including non-linear clearance described by a Michaelis-Menten term augmented with a time-dependent process to account for CD4 receptor kinetics changing after antibody administration (Fig. 2b and Methods). The PD was described by logistic model parameterized by the antibody binding *EC*_50_ and a slope. The final PK/PD model demonstrated good fit to the serum concentrations and CD4RO of the PLWoH groups (Fig. 2c and Table 3). The estimated two-compartment model PK parameters were consistent with those from other IgG antibodies with bioavailability of 63%^25–27^. Unfortunately, due to the limited sample size and PK complexity, attempts to include covariates were unsuccessful. The estimated serum clearance parameters capturing binding between 10E8.4/iMab and CD4 receptors indicated rapid binding kinetics (that is, high affinity). The velocity of CD4 receptor binding slowed following 10E8.4/iMab administration and stabilized <1 day. The estimated *EC*_50_ was 0.39 μg ml^−1^ near the lower limit of quantification (LLoQ) (0.2 μg ml^−1^), indicating binding rates accelerate at low serum concentrations. The final estimates for the CD4RO parameters were generally within an order of magnitude of those determined by previous PK models of iMab alone^27^.

**Table 3:** Population PK/PD estimates from the population fit to participants living without and with HIV who received 10E8.4/iMab

Parameter	Description	Estimate (relative standard error, %)		
		PLWoH	PLWH (base)	PLWH (covariate adjusted)
**Fixed effects (PK)**				
k_a_	Absorption rate (per day)	0.25 (18.09)		
F	Bioavailability	0.63 (16.03)		
V_c_	Volume, central compartment (liters)	2.38 (7.50)	3.07 (10.60)	2.68 (7.73)
βV,log(Wt)	Weight-adjustment covariate			0.97 (28.66)
Cl	Clearance (liters per day)	0.13 (17.91)	0.27 (21.29)	0.28 (14.39)
βCl,log(CD4pct)	CD4 percent-adjustment covariate			−0.59 (37.77)
Q	Intercompartmental clearance (liters per day)	0.43 (33.45)	0.26 (42.97)	0.31 (37.02)
V_p_	Volume, peripheral compartment (liters)	1.38 (14.06)	1.53 (31.24)	1.59 (20.23)
V_m,0_	Initial maximal velocity of the non-linear elimination (µg ml^−1^ day^−1^)	104.99 (70.05)		
v_k_	Exponential rate of change between V_m,0_ and V_m,∞_ (per day)	11.49 (181.23)		
V_m,∞_	Final maximal velocity of the non-linear elimination (µg ml^−1^ day^−1^)	19.95 (9.59)	10.22 (19.21)	10.06 (15.21)
K_m_	Concentration of half-maximal velocity (µg ml^−1^)	0.2	0.2	0.2
**Fixed effects (PD)**				
EC50	Concentration producing half occupation (µg ml−1)	0.39 (19.02)	0.34 (83.20)	0.38 (11.70)
h	Hill coefficient or slope	0.79 (3.04)	1.80 (51.70)	1.90 (8.67)
**Standard deviations of the random effects**				
ω_Vc_	Standard deviation, volume of central compartment	0.23 (14.61)	0.21 (45.39)	
ω_Cl_	Standard deviation, clearance	0.24 (39.87)	0.23 (28.12)	0.15 (28.40)
ω_EC50_	Standard deviation, *EC*_50_	0.85 (15.70)		
**Half-life**				
Approximate half-life (IV 10 mg kg^−1^)	Dose-dependent observed half-life during slowest, linear clearance (days)	3.4	2.5	2.3
Approximate half-life (IV 30 mg kg^−1^)	Dose-dependent observed half-life during slowest, linear clearance (days)	8.7	6.4	6.6
Maximum half-life	Theoretical half-life with fully saturated CD4 receptors (days)	21.3	13.7	12.3
**Error model parameters**				
σ_concentration_ (proportional)	Model standard error	0.21 (9.70)	0.26 (11.89)	0.26 (10.73)
*σ*_*CD4RO*_ (proportional)	Model standard error	0.50 (4.83)	0.15 (9.36)	0.15 (8.22)

PK parameters describe serum concentrations of 10E8.4/iMab. Standard errors for the PK model were estimated via linearization. Covariates for the PLWH model were centered on the median of the covariate distribution (70 kg for weight and 38.5% for CD4 percentage). *K*_*m*_ was fixed at 0.2 μg ml^−1^, the LLoQ. PD parameters describe CD4RO dynamics in the presence of 10E8.4/iMab.

Due to the non-linear PK, the elimination half-life of the bispecific antibody was not constant and was specifically dependent on CD4RO; however, the model predicted a maximal elimination half-life of 21.3 days under the conditions that either there are theoretically no free CD4 receptors or the serum concentrations are very large (approaching infinity). For the 10 mg kg^−1^ and 30 mg kg^−1^ groups, we estimated an approximate half-life corresponding to the slowest, linear clearance for these regimens while the CD4 receptors were highly occupied, a temporary phase dependent on the dose level. For group E, we estimated an approximate half-life of 3.4 days between days 3 and 10; for group F, we estimated an approximate half-life of 8.7 days between days 6 and 13 (Supplementary Fig. 2).

Using the model, we simulated a theoretical multi-administration trial in PLWoH: 3 doses administered 28 days apart with either 10 mg kg^−1^ SC or 30 mg kg^−1^ IV (Supplementary Fig. 3). The simulated trials highlight the complexities of designing a 10E8.4/iMab trial in contrast to other anti-HIV IgGs, specifically as CD4RO considerations must be incorporated to ensure steady concentrations are maintained. A computational tool was built to simulate trials under general design options (https://pk10e8imab.fredhutch.org/).

#### PK in PLWH

**Fig. 3:**
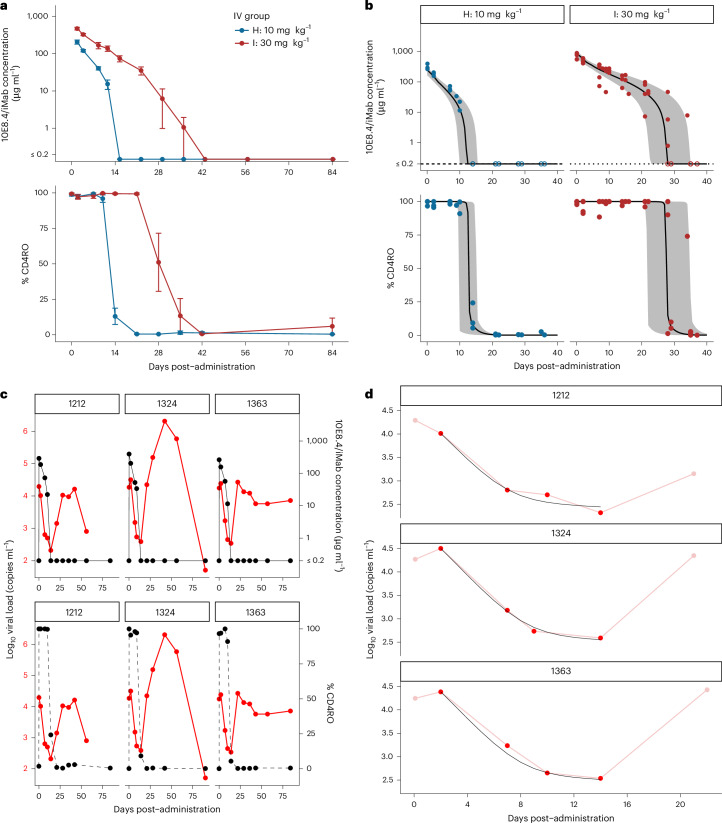
10E8.4/iMab serum concentration and percent CD4CD4RO levels by dose and route group (colors) among PLWH. **a**, Mean (lines) and standard errors (error bars) of observed serum concentrations over time (group H: *n* = 3, group I: *n* = 6). The first timepoint for the IV groups corresponds to 1 h after infusion. **b**, Population mean (lines) with 90% prediction interval (shaded region) using the fitted Michaelis-Menten (Fig. 2b) model for serum concentrations and CD4RO% with points representing observed data by dose and route group. **c**, Observed viral load concentration (red points, connected by red lines) among viremic participants in group H plotted over serum concentration and percent CD4RO. **d**, Viral load concentration measurements (copies per milliliter) from baseline (day 0) through first rebound timepoint, with theoretical curve (black line) based on combination ART viral suppression during viral decay phases. To match ART studies, for all three participants, a 2-day treatment effect lag period was applied, the first-phase and second-phase half-life were fixed to ART values of 0.67 and 25 days; respectively (Methods). The inflection point was fit by participant.

As among PLWoH, serum 10E8.4/iMab concentrations among PLWH exhibited non-linear PK concurrent with CD4RO kinetics as described by a TMDD PK system (Fig. 3a and Supplementary Table 1). The PK/PD model trained to PLWoH was initially applied to PLWH, but serum PK was significantly better captured when the model was fit directly to the PLWH cohort, reducing to a simpler Michaelis-Menten model without the time-dependent terms (corrected Bayesian information criterion (BICc) 1,018 versus 857; Supplementary Fig. 4). The final population PK/PD model, which included weight as a covariate on central volume and baseline CD4 percentage as a covariate on clearance, adequately captured the serum concentration and CD4RO data (Fig. 3b and Table 3). The estimated two-compartment model PK parameters were generally consistent with those from other anti-HIV IgG antibodies^25–27^.

10E8.4/iMab serum clearance (CL parameter) was approximately twofold faster among PLWH than PLWoH. We assessed whether this difference was attributable to differences in CD4 cell levels, but the covariate relationship observed among PLWH was insufficient to predict similar clearance levels among PLWoH (Supplementary Fig. 5). As a result, the maximal terminal half-life of 10E8.4/iMab for PLWH was 12.3 days, about twofold faster than PLWoH. Using the population PK parameters, for group H, we estimated an approximate half-life of 2.3 days between days 3 and 10; for group I, we estimated an approximate half-life of 6.6 days between days 7 and 14 (Supplementary Fig. 2).

We also observed the estimated PD slope (*h* parameter in Table 3) was steeper among PLWH, indicating that the increase and drop in CD4RO is faster among PLWH than PLWoH given the same serum antibody concentration, as the *EC*_50_ estimates were similar. The difference in slopes affects the concentrations required to achieve full occupancy. For PLWH, at 0, 1, 10, and 100 μg ml^−1^ of 10E8.4/iMab, the model predicts concurrent 22.7%, 86.1%, 99.8% and 100% CD4RO, respectively. Conversely, to achieve 50%, 80%, 99% and 99.9% CD4RO, concurrent concentrations of 10E8.4/iMab must reach 0.34 (the *EC*_50_), 0.79, 4.3 and 15 μg ml^−1^, respectively. For PLWoH, the model suggested that much higher concentrations are required to achieve such occupancy (for example, 130 μg ml^−1^ to achieve 99% occupancy).

#### Antiviral activity

HIV plasma viral load (VL) was measured in the three group H participants (PLWH receiving 10 mg kg^−1^) (Fig. 3c and Extended Data Table 3). Participants had similar VL measurements at baseline (17,600–19,600 copies ml^−1^). Each participant’s VL fell sharply following 10E8.4/iMab administration and rebounded as the antibody concentration and CD4RO decreased to low levels. Nadir VL was observed on day 14 for all three participants, reaching similar levels (209–389 copies ml^−1^). The estimated participant-specific VL overall reduction ranged between 1.68 and 1.97 logs. No mutations associated with 10E8.4/iMab resistance emerged in the rebound virus (Supplementary Table 2). We next predicted antiviral activity based on combination ART as applied to the observed VL trajectories (Methods). Assuming a 2-day lag before antiviral effect, we observed that the theoretical ART VL trajectories were remarkably consistent with these data until VL rebound, which is suspected to have occurred between study days 14 and 21 (Fig. 3d).

#### ADAs

**Extended Data Fig. 4:**
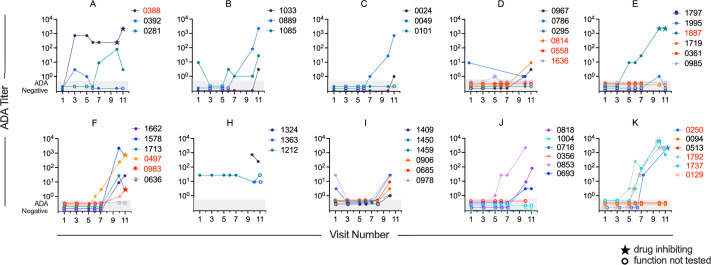
Anti-drug antibody (ADA) responses over time. ADA titers over time per 10E8.4/iMab treated participant (colors) by study group **(A**-**K**). Each symbol represents a unique sample that was evaluated for ADA in the bridging assay. Samples that were ADA negative are graphed in the gray region of each graph. Functional inhibition of 10E8.4/iMab neutralizing activity was evaluated in a subset of samples: those in which it was detected (star), not detected (filled circle), or not evaluated (hollow circle) are indicated. PubIDs in red indicate participants in whom functional ADA were detected at one or more timepoints.

ADAs were detected in all study groups during follow-up among participants receiving 10E8.4/iMab (Extended Data Table 4 and Extended Data Fig. 4). Treatment-induced or boosted ADA responses were observed in 25/39 (64%) PLWoH and 5/7 (71%) PLWH (with two ADA-positive PLWH missing baseline sample results needed to determine treatment effect). Consistent with the time required to induce a response and the ability of circulating drug to reduce ADA assay sensitivity, most treatment-related ADA responses were detected later in the study (after day 28, all PLWH at day 84), at which point 10E8.4/iMab levels in the sera were undetectable. Although treatment-induced ADAs were observed as early as the first post-infusion timepoint tested (visit 3), ADA response rate and magnitude generally increased over time (Extended Data Fig. 4). No clear ADA trends associated with 10E8.4/iMab route, dose or HIV status were observed, though the high overall response rate and small study size limited power to investigate these factors. There were no ADA-related safety concerns (for example, hypersensitivity reactions or CD4^+^ T cell depletion).

ADA functional relevance was evaluated by defining the 10E8.4/iMab inhibiting activity of a selected subset of serum samples. Based on ADA positivity in the binding assay, 40 participants were selected for evaluation of reduced 10E8.4/iMab neutralizing activity in vitro, with reduction observed in 11 of these participants (Extended Data Fig. 4 and Supplementary Table 3). Among these, inhibition of 10E8.4/iMab function was detected in 10/30 participants with definitively treatment-boosted or treatment-induced ADA and in one participant (PubID 0558) in whom an ADA response was not detected at any timepoint in the bridging assay (Extended Data Fig. 4 and Supplementary Table 3). Drug inhibition was typically observed in participants that exhibited high and increasing ADA titers, though many samples with high titers did not show inhibitory activity, and some samples that had only low titers (for example, PubID 1636) or were ADA negative exhibited some inhibition of 10E8.4/iMab-mediated neutralization (Extended Data Fig. 4).

**Extended Data Fig. 5:**
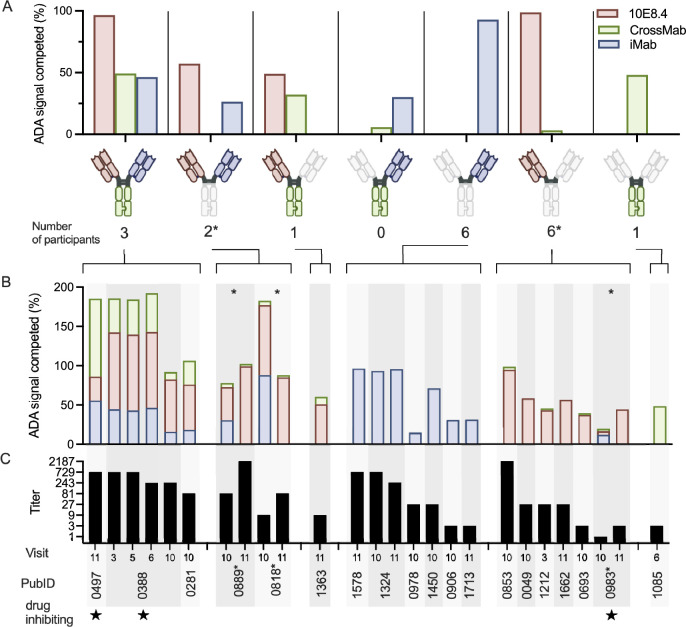
Epitope-specificity of ADA responses. **a**. Representative plots showing the percentage of ADA signal competed by incubation in the presence of either 10E8.4 (red), CrossMab (green), or iMab (blue). Samples were categorized by visual inspection as either comprising ADA toward a single or multiple domains as evidenced by the degree of signal suppression observed for each competitor molecule. The number of participants exhibiting each profile is listed below its graphical representation. **b**. Stacked bar graph of ADA signal competition results for each mapped sample. Asterisks indicate participants whose epitope mapping profiles changed over time. **c**. Titers and positive functional drug inhibition activity (star) for each mapped sample.

Given this striking rate of immunogenicity, samples from a subset of 21 participants in whom bridging ADAs were detected, selected based on sample availability, were epitope mapped to define the sites recognized by ADA. Competition with conventional monoclonal antibody forms of 10E8.4, iMab and an unrelated bispecific containing the modified Fc ‘CrossMab’ substitutions was used to identify immunogenic epitopes (Extended Data Fig. 5a). The most common ADA profile observed targeted either the 10E8.4 or the iMab arm, although numerous participants also had ADA compositions that targeted multiple domains (Extended Data Fig. 5b). When multiple timepoints for a given participant were evaluated, ADA composition often remained relatively consistent (for example, PubIDs 0388 and 1324) (Extended Data Fig. 5c). However, three participants (PubIDs 0889, 0818 and 0983) demonstrated a competition profile dominated by 10E8.4 by the final timepoint tested while showing iMab reactivity at a prior timepoint. Among the set of participants whose ADA responses were epitope mapped, three demonstrated drug inhibition. Of these, two had ADA targeting all three 10E8.4/iMab domains, and one presented evidence of only 10E8.4-specific ADA.

**Extended Data Fig. 6:**
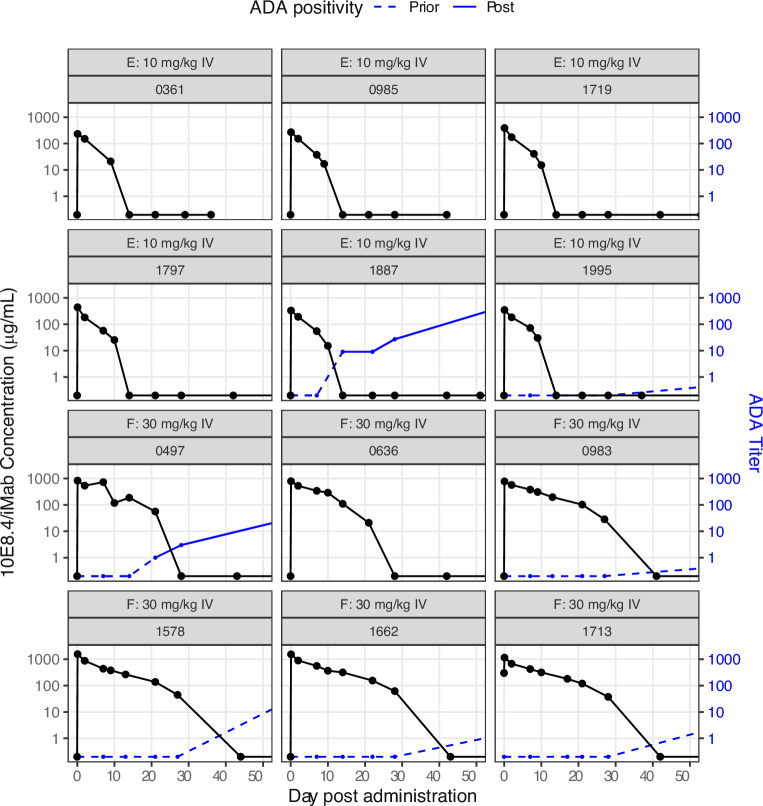
ADA titers overlaid with 10E8.4/iMab serum concentrations. ADA binding titers (blue) overlaid with 10E8.4/iMab serum concentrations (black) among PLWoH receiving 10 mg kg^−1^ or 30 mg kg^−1^. ADA measurements were obtained at Visit 1 (Day 0), Visit 3 (Day 7), Visit 5 (Day 14 ± 1), Visit 6 (Day 21 ± 1), Visit 7 (Day 28 ± 1), Visit 10 (Day 84 ± 7), and Visit 11 (Day 168 ± 14).

Finally, we visually assessed concurrent positive serum concentration and ADA titers among PLWoH receiving 10 mg kg^−1^ IV or 30 mg kg^−1^ IV (where there were multiple, albeit limited, concurrent observations); we observed no evidence that trajectories varied by ADA status (Extended Data Fig. 6).

## Discussion

This trial demonstrates the safety and tolerability of a novel bispecific antibody when given intravenously in PLWH and PLWoH and subcutaneously in PLWoH. 10E8.4/iMab was safe and well tolerated across dose levels and administration routes. Laboratory findings were notable for ADA development once 10E8.4/iMab concentrations declined. Although the rate of ADA detection, the multiple epitopes targeted and the ability of some ADA responses to inhibit drug function suggest high immunogenicity of this molecule, no related AEs, and notably no CD4^+^ T cell depletion, were seen among participants with ADA.

This study was conducted partially during the COVID-19 pandemic, with attendant challenges and successes. Amendments to the protocol to facilitate study completion, specifically with regard to decreasing the enrollment of PLWH with active viremia, limited our ability to draw conclusions about 10E8.4/iMab antiviral properties. Despite the pandemic, participant retention was excellent.

The PK model describing 10E8.4/iMab serum concentrations was more complex than other anti-HIV IgG antibodies owing to the interaction of the iMab arm and host CD4 receptors. However, shared mechanisms between 10E8.4/iMab and other HIV antibodies had similar parameter estimates, indicating inheritance of IgG clearance mechanisms when not bound to CD4 receptors^25,26^. The final model for PLWoH included a time-varying elimination process, potentially reflecting temporal reduction in CD4 molecules on CD4^+^ T cells after 10E8.4/iMab exposure (as seen with iMab alone)^28^ and altered rate of iMab arm binding as CD4RO increases. Interestingly, this process did not explain the PK observed among PLWH, which may indicate different CD4 receptor kinetics or a more rapid effect (that is, within hours) not captured in the data. The remaining estimated parameters appeared generally similar between the PLWoH and PLWH models. Two key differences emerged: 1) elimination independent of CD4RO was about twofold faster in PLWH, and 2) full CD4RO was achieved at lower antibody concentrations (>10-fold) in PLWH. Theoretical half-life calculations illustrated the effects of faster linear clearance. The maximal terminal half-life—achieved in the absence of CD4 receptors or when serum concentration approached infinity—was 12 days in PLWH versus 21 days in PLWoH. Differences in CD4 cell count between the populations did not explain differences in clearance. Although this finding should be interpreted cautiously given the small number of PLWH enrolled here, faster bnAb elimination in PLWH has been observed previously in multiple clinical trials and attributed to higher antigen-dependent accelerated clearance among PLWH^29–33^. Estimated CD4 receptor binding rates were lower in PLWH (indicated by lower non-linear elimination velocity in the Michaelis-Menten term), potentially suggesting a lower pool of receptors (for example, due to downregulation of CD4 expression)^34^ or slower receptor turnover. Differences in parameter estimates may also reflect unmeasured HIV binding sources affecting all three processes. The model complexities posed calibration challenges. Although weight commonly affects PK, we could not adjust for covariates in the PLWoH model given the sample size. Confident application of the model results is thus limited to similar populations; extrapolations (for example, trial simulations) should be cautiously interpreted.

Notably, 63% of participants developed ADAs, including 23% with elevated functional ADA ID50 titers. These are higher ADA response rates than frequently observed with HIV antibodies^35–37^. Based on our epitope mapping, 10E8.4-specific and iMab-specific ADAs were observed with similar frequency. Although not comprehensive, these data do not suggest that 10E8.4 was particularly immunogenic regarding ADA development. Interestingly, the CrossMab component appeared to be the least frequent target of ADA specificity. Although there was no evidence that PK/PD varied by ADA status, data were limited to make this comparison, as many participants cleared 10E8.4/iMab before ADAs developed. Rapid clearance of a biologic in advance of ADA detection is commonly observed^38,39^; here, sampling density and sample size precluded principled analysis to understand whether ADA affected PK/PD. Subsequent clinical trials of 10E8.4/iMab should continue to evaluate ADA epitope specificity and include repeated 10E8.4/iMab doses to clarify whether ADA responses might persist or strengthen, affect PK, and/or pose a barrier to clinical development.

The biphasic antiviral activity predicted under ART is based on the assumption that ART completely interrupts viral replication, even though low-level transcription and translation from an active reservoir can persist despite ART^40–42^. Both arms of the bispecific antibody—10E8.4 binding to membrane-proximal external region and iMab causing CD4RO—could disrupt viral replication, but the individual contribution of each arm of the bispecific antibody remains unclear. Although the curves derived using ART-based estimates recapitulated the VL trajectories measured in this study, we should not exclude 10E8.4/iMab antiviral mechanisms that differ from conventional ART. 10E8.4/iMab was engineered to have low Fc-effector functions (for example, antibody-dependent cell-mediated cytotoxicity and complement-dependent cytotoxicity), but limited residual antiviral activity could cause steeper viral decline; we cannot exclude a biphasic curve with a steep first phase lasting 1 day. Alternatively, the first phase may be up to twofold slower than under ART, based on the log-linear decay observed between days 2 and 7 here^43,44^. A slower clearance scenario could suggest partial effectiveness, and the second phase would not be solely explained by long-lived infected cells. More frequent sampling (for example, daily sampling in the first week) in future studies could elucidate this. Of note, the antiviral effects observed were similar to previous study of PLWH with PGT121-sensitive viruses who received an administration of PGT121 (1.77 logs)^29^. However, all antiviral conclusions here are preliminary given the low number of participants with active viremia. Notably, no mutations likely to confer resistance to either arm of 10E8.4/iMab emerged in rebound virus among participants with active viremia, although this was measured only among three participants.

These data have implications for future clinical trials of 10E8.4/iMab. Our finding that much higher concentrations of 10E8.4/iMab were required to achieve a given level of CD4RO among PLWoH compared to PLWH can inform future trial design with iMab-based products and other post-attachment entry inhibitors; different dosing strategies may be required depending on the study population. Here, we developed a novel PK/PD model and corresponding tool that can inform future dosing (https://pk10e8imab.fredhutch.org/) and trial design for combination studies of 10E8.4/iMab and other bnAbs. Notably, 10E8.4/iMab is currently under study alone and in combination with VRC07-523LS (a CD4 binding site bnAb) among PLWH in Tanzania in a phase 1b single-dose trial (RV584, NCT05890963). Future studies can elucidate whether 10E8.4/iMab PK changes significantly with repeat dosing, particularly after ADA development. ADA data from RV584 and future studies of 10E8.4/iMab (delivered IV or SC) will be critical to understanding whether 10E8.4/iMab has a role in HIV treatment or is better suited for short-term HIV prevention (for example, peri-exposure prophylaxis in situations where daily oral ART is infeasible).

This study has limitations. As described above, denser ADA sampling could have supported analysis of potential ADA impact on PK. Including more participants with active viremia could have enhanced antiviral analyses and surveillance for mutations conferring 10E8.4/iMab resistance in rebound virus. Although the trial reached its goal of enrolling at least approximately 40% of each sex overall, there were no female PLWH. Women are underrepresented in HIV research; additional investment is warranted to support their clinical trial engagement^45–47^.

Our findings support the use of a post-attachment entry inhibitor coupled with one or more bnAbs targeting the HIV viral envelope. Another trial recently showed that every-eight-week infusions of a second-generation post-attachment entry inhibitor (which, like iMab, binds the CD4 receptor) along with VRC07-523LS maintained viral suppression for 24 weeks among PLWH^48^. Taken together with our findings, these data provide proof of concept for this complementary approach. Safety and tolerability data from ABA-0101 provide rationale for further investigation of 10E8.4/iMab. These investigations contribute to ongoing efforts to expand the pool of emerging options for HIV treatment and prevention, and in doing so, to end the epidemic.

## Methods

### Ethics

This study complied with all relevant ethical regulations. The study and all amendments and protocol deviations were approved by the Institutional Review Board of Columbia University Irving Medical Center (CUIMC; IRB AAAS1239) and the Advarra Institutional Review Board (IRB PRo00037972). The study was overseen by an independent Safety Monitoring Committee, which provided an ongoing assessment of participant safety during the conduct of the study. The Safety Monitoring Committee consisted of three independent individuals who had no relationship to the principal investigator and co-investigators involved in the trial and who had no direct responsibility for the clinical care of study participants. All participants provided written informed consent. The Community Advisory Board of the Columbia Collaborative Clinical Trials Unit provided input regarding community engagement and trial conduct. This study was registered at ClinicalTrials.gov (number NCT03875209) on 14 March 2019.

### Study design and participants

ABA-0101 was a phase 1, partially randomized, dose-escalation study conducted at two centers in the US (CUIMC in New York and Orlando Immunology Center in Florida) between 21 March 2019 and 1 October 2021. The primary aims were to evaluate the safety and tolerability profile of a single intravenous infusion of 10E8.4/iMab at 5 dose levels (0.3 mg kg^−1^, 1 mg kg^−1^, 3 mg kg^−1^, 10 mg kg^−1^ and 30 mg kg^−1^) among PLWoH, a single intravenous infusion of 10E8.4/iMab at two dose levels (10 mg kg^−1^ and 30 mg kg^−1^) among PLWH, and a single subcutaneous injection of 10E8.4/iMab at 3 dose levels (1 mg kg^−1^, 2.5 mg kg^−1^ and 10 mg kg^−1^) among PLWoH (Fig. 1, Consort Diagram).

Risk reduction counseling was conducted for all participants throughout the study. Transmission risk reduction counseling for PLWH not on ART included counseling regarding condom use and linkage of potential partners to biomedical HIV prevention (for example, PrEP and PEP) care.

### Eligibility criteria are presented below

**Inclusion criteria for PLWoH:**

1. Healthy volunteers born male and female as assessed by medical history and physical examination
2. Aged ≥18 and ≤60 years at the time of screening
3. Ability and willingness to provide written informed consent
4. Willingness to comply with protocol schedule
5. Willingness to undergo HIV-1 testing
6. Non-reactive fourth-generation point of care HIV-1 test at screening
7. Hepatitis B surface antigen negative
8. Hepatitis C antibody negative, or if reactive, hepatitis C RNA undetectable in plasma
9. Volunteers born female of reproductive potential who are sexually active with a male sex partner must agree to use one effective method of contraception from the time of signing the consent to completion of the study and agree to pregnancy testing as per the schedule of events.
10. Study participants born female with reproductive potential are defined as premenopausal volunteers born female who have not had a sterilization procedure (for example hysterectomy, bilateral oophorectomy, tubal ligation or salpingectomy). Volunteers born female are considered menopausal if they have not had a menses for at least 12 months and have a follicle-stimulating hormone (FSH) of greater than 40 IU liter^−1^ or if FSH testing is not available, they have had amenorrhea for 24 consecutive months.

**Exclusion criteria for PLWoH:**

1. Confirmed HIV-1 infection
2. Weight above 130 kg at screening. Note that subjects above 80 kg may not be randomized into the 2.5 and 10 mg kg^−1^ SC dosing groups (arm 4 groups J and K).
3. At high risk of HIV-1 infection as defined by one of the following:
  - Unprotected intercourse with a casual or HIV-infected partner over the past 12 months
  - In a serodisconcordant relationship with an HIV-1 infected partner
  - A diagnosis of a new sexually transmitted infection within the past 12 months
  - Exchange of money or drugs for sex in the last 12 months
  - More than 2 sexual partners, defined as insertive or receptive vaginal or anal intercourse, within the past 6 months
4. Any acute or chronic medical condition that in the opinion of the investigator would preclude participation
5. Immunodeficiency or chronic autoimmune disease
6. IV drug use
7. Excessive use of alcohol or recreational drugs that in the opinion of the investigator would preclude participation.
8. Decompensated psychiatric illness
9. Need for chronic immunotherapy including systemic corticosteroids, other monoclonal antibody therapy or immunosuppressive drugs
10. Volunteers born female who are pregnant, lactating or planning on becoming pregnant over the study period
11. Any of the following laboratory parameters:
  - Hemoglobin <10.0 g dl^−1^
  - Absolute neutrophil count <1,000 mm^−^^3^
  - Absolute lymphocyte count <500 mm^−^^3^
  - Platelet count <100,000 mm^−3^
  - Prothrombin time > 1.25× upper limit of normal (ULN)
  - Partial thromboplastin time > 1.66× ULN
  - Creatinine >1.25× ULN
  - Aspartate aminotransferase > 1.5× ULN
  - Alanine transaminase > 1.5× ULN
  - Glucose (non-fasting) >160 mg dl^−1^
  - Proteinuria: 2+ or greater
  - Hematuria: >10 red blood cells (RBCs) per high-power field
  - Serum calcium < 8.5 mg dl^−1^ or >10.2 mg dl^−1^
  - Serum PTH levels <10 pg ml^−1^ or >65 pg ml^−1^
12. Any vaccine administration within 14 days of study entry
13. Experimental HIV-1 vaccine in past (active arm of an HIV-1 vaccine trial if applicable)
14. Previous receipt of an experimental mAb to HIV-1 in a research study
15. History of severe allergic reactions to drugs, vaccines, or drug infusion
16. Participation in another investigational clinical trial within the past 12 weeks or anticipated during the course of the current study

**Inclusion criteria for PLWH arm 3 group H:**

1. Aged ≥18 and ≤60 years at the time of screening
2. Ability and willingness to provide written informed consent
3. Willingness to comply with protocol schedule
4. Willingness to undergo HIV-1 testing
5. Reactive fourth-generation point of care HIV-1 test at screening
6. Plasma HIV-1 RNA levels ≥ 1,000 copies ml^−1^ and ≤ 100,000 copies ml^−1^ in subjects who are either:
  - ART-naïve
  - ART-experienced and in consultation with their primary provider have discontinued therapy for at least 4 weeks
  - ART-experienced, clinically stable and without changes to their ART regimen for at least 4 weeks
7. CD4^+^ T cell count >350 cells mm^−^^3^ and nadir CD4^+^ T cell count ≥250 cells mm^−3^
8. Agrees not to begin or change antiretroviral therapy for 6 weeks after 10E8.4/iMab infusion despite a clear explanation of current DHHS guidelines
9. Hepatitis B surface antigen negative
10. Hepatitis C antibody negative or if reactive hepatitis C RNA undetectable in plasma
11. Volunteers born female of reproductive sexually active with a male sex partner potential agree to use an effective method of contraception from the time of signing consent to the end of the study and agree to pregnancy testing as per the schedule of events.
12. Study participants born female with reproductive potential are defined as premenopausal volunteers born female who have not had a sterilization procedure (for example hysterectomy, bilateral oophorectomy, tubal ligation or salpingectomy). Volunteers born female are considered menopausal if they have not had a menses for at least 12 months and have a FSH of greater than 40 IU liter^−1^ or if FSH testing is not available, they have had amenorrhea for 24 consecutive months.

**Inclusion Criteria for PLWH arm 3a group I:**

1. Aged ≥18 and ≤60 years at the time of screening
2. Ability and willingness to provide written informed consent
3. Willingness to comply with protocol schedule
4. Willingness to undergo HIV-1 testing
5. Reactive fourth-generation point of care HIV-1 test at screening
6. Plasma HIV-1 RNA below the level of detection at screening and documented within 3 months of screening
7. On suppressive triple-combination antiretroviral therapy as per DHHS guidelines
8. Current CD4^+^ T cell count >350 cells mm^−3^
9. Agrees not to change antiretroviral therapy for 6 weeks after 10E8.4/iMab infusion
10. Hepatitis B surface antigen negative
11. Hepatitis C antibody negative or if reactive hepatitis C RNA undetectable in plasma
12. Volunteers born female of reproductive potential, sexually active with a male sex partner agree to use one effective method of contraception from the time of signing the consent to completion of the study and agree to pregnancy testing as per the schedule of events.
13. Study participants born female with reproductive potential are defined as premenopausal volunteers born female who have not had a sterilization procedure (for example hysterectomy, bilateral oophorectomy, tubal ligation or salpingectomy). Volunteers born female are considered menopausal if they have not had a menses for at least 12 months and have an FSH of greater than 40 IU liter^−1^ or if FSH testing is not available, they have had amenorrhea for 24 consecutive months.

**Exclusion criteria for PLWH:**

1. Any acute or chronic medical condition that in the opinion of the investigator would preclude participation
2. A history of virologic failure of two or more combination ART regimens. A regimen switch due solely to intolerance and not virologic failure does not qualify as a failed regimen.
3. In arm 3 group H, weight above 130 kg and arm 3a group I weight above 100 kg at the time of screening
4. Excessive use of alcohol or recreational drugs that in the opinion of the investigator would preclude participation
5. Decompensated psychiatric illness
6. Need for chronic immunotherapy including systemic corticosteroids, other monoclonal antibody therapy or immunosuppressive drugs
7. Volunteers born female who are pregnant, lactating or planning on becoming pregnant over the study period
8. Any of the following laboratory parameters
  - Hemoglobin <10.0 g dl^−1^
  - Absolute neutrophil count <1,000 mm^−^^3^
  - Absolute lymphocyte count <500 mm^−^^3^
  - Platelet count <100,000 mm^−3^
  - Prothrombin time > 1.25× ULN
  - Partial thromboplastin time > 1.66× ULN
  - Creatinine >1.25– ULN
  - Aspartate aminotransferase > 1.5× ULN
  - Alanine transaminase > 1.5× ULN
  - Glucose (non-fasting) >160 mg dl^−1^
  - Proteinuria: 2+ or greater
  - Hematuria: >10 RBCs per high-power field
  - Serum calcium < 8.5 mg dl^−1^ and >10.2 mg dl^−1^
  - Serum PTH level <10 pg ml^−1^ or >65 pg ml^−1^
9. Any vaccine administration within 14 days of study entry
10. Experimental HIV-1 vaccine in past (active arm of an HIV-1 vaccine trial if applicable)
11. Participation in a research study of a neutralizing mAb to HIV-1
12. History of severe allergic reactions to drugs, vaccines, or drug infusion
13. Participation in another investigational clinical trial within the past 12 weeks or anticipated during the course of the current study (allowed is participation in a clinical trial or observational study that did not include receipt of investigational product)

Enrollment was underway when the COVID-19 pandemic began in the United States in 2020. Participants were not enrolled between 6 February 2020 and 14 June 2020, as clinical and research efforts were directed toward the initial pandemic response; follow-up visits continued as scheduled.

### Protocol amendments

There were eight amendments to the original protocol.

Protocol Amendment 1 was issued on 14 January 2019. The overall reason for the amendment was to incorporate feedback from the US Food and Drug Administration. The main changes were to add a 0.3 mg kg^−1^ initial IV dosing group to arm 1 for additional safety evaluation, to add a safety lab timepoint at day 7 for additional safety monitoring, to exclude PLWH who had experienced virologic failure on at least 2 or more previous treatment regimens as they may require the use of iMab in the future to construct an effective ART regimen, to augment PK analyses by adding a timepoint for collection of levels of circulating and CD4-bound 10E8.4/iMab (one hour after the end of 10E8.4/iMab administration), and to change attribution of AE relatedness to ‘related’ or ‘not related’ (previously categorized as ‘possibly’, ‘probably,’ or ‘definitely’ related) based on updated Food and Drug Administration guidelines.

Protocol Amendment 2 was issued on 21 February 2019. The overall reason for the amendment was to update study procedures. The main changes were to adjust the maximum allowable volume in one blood draw to align with the laboratory assays planned and to add the update that 10E8.4/iMab would be shipped to clinical sites from a designated Current Good Manufacturing Practice storage facility rather than be shipped directly from the manufacturer.

Protocol Amendment 3 was issued on 25 July 2019. The overall reason for the amendment was to accelerate the enrollment schedule based on early safety and tolerability data. Instead of requiring each group to enroll sequentially, groups were combined into sequences that allowed two groups to enroll simultaneously; a participant who was eligible for both open groups could choose the group into which they enrolled (Fig. 1b). The timing of Safety Monitoring Committee meetings was adjusted accordingly. In addition, the requirement for hepatitis B and C screening was adjusted, so that participants who had an initial screening visit (including hepatitis B and C screening) more than 28 days before enrollment could defer repeat hepatitis B and C testing at the time of their rescreening, if the previous hepatitis testing was performed within 90 days of enrollment.

Protocol Amendment 4 was issued on 16 October 2019. The overall reason for the amendment was to add a statement regarding potential replacement of subjects who do not complete study visits after receiving 10E8.4/iMab. The main change was to add that if a participant was lost to follow-up within 4 weeks of receiving 10E8.4/iMab, an additional subject could be added to that Study arm and Dosing group.

Protocol Amendment 5 was issued on January 8, 2020. The overall reason for the amendment was to respond to early findings of high prevalence of ADA in arm 1 participants. The main changes were to add T cell subset monitoring for all study participants (previously T cell subsets were planned to be monitored only among PLWH) and to add additional ADA collection timepoints.

Protocol Amendment 6 was issued on 25 February 2020. The overall reason for the amendment was in response to the challenges of identifying PLWH with viremia who were eligible for enrollment. The main changes were to remove arm 3 group G (3 mg kg^−1^ IV, PLWH with viremia; *n* = 6) based on the low expectation of seeing antiviral activity at this dose and given preliminary safety data from already enrolled groups supporting higher dosing and to adjust the exclusion criteria for PLWH so that ‘intravenous drug use’ was not exclusionary (instead maintaining the exclusion criterion ‘Excessive use of alcohol or recreational drugs that in the opinion of the investigator would preclude participation’).

Protocol Amendment 7 was issued on 24 August 2020. The overall reason for this amendment was to respond to ongoing challenges associated with enrolling PLWH with viremia after the onset of the pandemic. The main changes were 1) to adjust the sample size for PLWH, so that arm 3 groups H and I would enroll four participants each instead of six participants each; 2) adjust the inclusion criteria to allow a lower HIV-1 RNA at enrollment (greater than or equal to 1,000 copies ml^−1^ rather than 2,000 copies ml^−1^) and allow a shorter period of time during which participants were off ART or on stable ART before enrollment (four weeks instead of 8 weeks); and 3) add an additional timepoint for PK assays and HIV-1 RNA.

Protocol Amendment 8 was issued on 25 March 2021. The overall reason for the amendment was in response to persistent challenges enrolling PLWH with viremia. The main changes were 1) to reduce the number of participants in arm 3 group H (10 mg kg^−1^ IV, PLWH with viremia) from four to three; and 2) to adjust arm 3 group I (30 mg kg^−1^ IV, PLWH with viremia) to arm 3a group I (30 mg kg^−1^ IV, PLWH with viral suppression) with a group size of 6 participants.

### Statistical analysis

The study was not powered for formal statistical hypothesis testing. Statistical analysis of study data was planned to be primarily descriptive, with emphasis placed on tabular and graphical displays. Statistical analysis was performed in R (v4.4.0) using tidyverse (v2.0.0) unless stated otherwise^49,50^.

The sample size per group, including the changes in the amendments described above, was determined based on the probabilities of observing AEs. The number of participants per group who received an administration of 10E8.4/iMab varied from 3 to 6, depending on group assignment. Assessment of injection site reaction dose-limiting toxicity in arm 4 (subcutaneous dosing) was based on a group size of 9 (6 active and 3 placebos). The power analysis therefore considered groups of size *n* = 3, *n* = 6, and *n* = 9 to understand the probability of observing AEs when the true event rate ranged from 1% to 30%. The safety and tolerability analysis was a modified intent-to-treat analysis in that individuals who received a group assignment but did not receive 10E8.4/iMab or placebo did not contribute data and hence were excluded from subsequent analysis. Descriptive analysis of safety and tolerability data was performed using SAS 9.4.

Solicited AE data were collected after 10E8.4/iMab administration. The number and percentage of participants experiencing each type of solicited sign or symptom was tabulated by severity. For a given sign or symptom, each solicited AE per participant was counted once under the maximum severity for all assessments.

Unsolicited AEs were coded into MedDRA preferred terms. The number and percentages of participants experiencing each specific AE was tabulated by severity and relationship to treatment. Each unsolicited AE per participant was counted once under the maximum severity or strongest recorded causal relationship to 10E8.4/iMab.

To ensure that male and female participants would be adequately represented in the study, the protocol stipulated that the trial would seek to enroll at least approximately 40% of each sex assigned at birth overall. Sex and gender were determined based on participant self-report. No sex- or gender-based analyses were planned, as the small number of participants overall was insufficient to enable meaningful sub analyses.

Randomization was performed for all participants in arm 4. PLWoH in arm 4 group J were randomized to receive 2.5 mg kg^−1^ 10E8.4/iMab SC or placebo SC in a 2:1 ratio (using 3 blocks of size 3). PLWoH in arm 4 group K were randomized to receive 10 mg kg^−1^ 10E8.4/iMab SC or placebo SC in a 2:1 ratio (using 3 blocks of size 3). Randomization was performed using SAS 9.4 by the Data Coordinating Center. Study staff (including clinical and laboratory staff) and participants were blinded to the treatment assignment; the study pharmacist at each site was unblinded. Unblinding was performed once all participants completed the day 84 visit.

### Study procedures

#### Clinical procedures

Potential participants were screened for eligibility, completed the informed consent process, and were enrolled within 28 days of screening. Participants were enrolled sequentially upon meeting study criteria in a dose-escalation schema with prespecified safety review by the Safety Monitoring Committee to allow progression to later dosing groups. Study participants were first enrolled in arm 1 group A (0.3 mg kg^−1^ IV among PLWoH), followed by arm 1 group B (1 mg kg^−1^ SC among PLWoH) and then by arm 1 group C (1 mg kg^−1^ IV among PLWoH). Participants were then enrolled in sequences. Sequence 1 comprised arm 4 group J (2.5 mg kg^−1^ SC or placebo among PLWoH) and arm 2 group D (3 mg kg^−1^ IV among PLWoH). Sequence 2 comprised arm 4 group K (10 mg kg^−1^ SC or placebo among PLWoH) and arm 2 group E (10 mg kg^−1^ IV among PLWoH). Sequence 3 comprised arm 2 group F (30 mg kg^−1^ IV among PLWoH) and arm 3 group H (10 mg kg^−1^ IV among PLWH with active viremia). Sequence 4 comprised arm 3a group I (30 mg kg^−1^ IV among PLWH with viral suppression).

In the IV dosing groups, 10E8.4/iMab was administered via a peripheral vein in an upper extremity over 60 min as an IV infusion, and the IV line was flushed with normal saline at the end of the infusion to ensure all product was delivered. In the SC dosing groups, 10E8.4/iMab was administered either as a single injection (1 mg kg^−1^, 2.5 mg kg^−1^) or as up to 4 SC injections not to exceed 2.0 ml per injection (10 mg kg^−1^) in the upper extremities, lower extremities (thigh) or abdomen.

Study participants were closely observed for 60 min after 10E8.4/iMab infusion/injection, with monitoring of vital signs and collection of solicited AEs. Participants completed diary cards capturing solicited systemic and local AEs for 14 days after receipt of 10E8.4/iMab. Solicited systemic AEs included fever, chills, fatigue, headache, nausea, rash, pruritus, stomach pain, diarrhea, vomiting, malaise, myalgia, lymphadenopathy and arthralgia. Solicited local AEs included local infusion/injection site reactions such as pain, tenderness, swelling, erythema, induration, warmth, itching at the infusion/injection site, nodule formation and bruising at the infusion/injection site. Unsolicited AEs were documented throughout the duration of the study.

The DAIDS AE Grading Table v2.1 was used to grade AEs occurring in arm 3 (enrolling PLWH) except those related to infusion reactions and cytokine release syndromes. The Toxicity Grading Scale for Healthy Adult and Adolescent Volunteers Enrolled in Preventive Vaccine Clinical Trials (2007) was used to grade AEs in arms 1, 2 and 4 (enrolling PLWoH)except those related to infusion reactions and cytokine release syndromes. The Common Terminology Criteria for Adverse Events (CTCAE) v. 5.0 grading scale was used for reporting and grading AEs related to infusion reactions and cytokine release syndromes.

Laboratory monitoring for safety assessment (complete blood count with differential, coagulation studies, parathyroid hormone level, chemistry profile, urinalysis, pregnancy testing and HIV testing) was performed at prespecified timepoints up to and including the last study visit, which was performed approximately 168 days after study enrollment.

#### Laboratory procedures

HIV-1 VL was measured among PLWH before 10E8.4/iMab administration and at prespecified timepoints throughout the study. Standard HIV-1 VL measurement, with an assay detection range of 20 copies ml^−1^, was performed at a CLIA-certified laboratory (at CUIMC for participants enrolled there, and at Quest Diagnostics for participants enrolled at Orlando Immunology Center). CD4^+^ T cell counts and percentages (CD4^+^ cells as a percentage of total lymphocytes) were measured using a clinical flow cytometry assay performed at CUIMC or Quest Diagnostics, according to site of enrollment.

10E8.4/iMab levels, ADA and CD4RO levels were measured among all participants before 10E8.4/iMab administration and at prespecified timepoints throughout the study.

An enzyme-linked immunosorbent assay (ELISA) was used to measure unbound 10E8.4/iMab levels through the quantitative antibody sandwich enzyme immunoassay technique. ELISA plates were coated with human CD4/LEU3 protein, followed by washing and blocking. The calibrators, quality controls, and samples were then added to the wells. Following incubation, the plates were washed and mouse-derived 10E8.4 anti-idiotype antibody was added to the wells. Subsequently, after further incubation, the plates were washed, and HRP-anti-mouse IgG was added to the wells. After further incubation, plates were again washed, and tetramethyl benzidine substrate was added. After sufficient color development, the reaction was stopped by the addition of stop solution, and the optical density of each well was determined by wavelength measurement at 450 nm with a correction at 630 nm. Signal data were analyzed using SoftMax Pro GxP software. A standard curve was generated for each plate, and the sample concentrations were interpolated from the plot.

To measure the presence and magnitude of ADAs capable of bridging drug, separate aliquots of 10E8.4/iMab were covalently conjugated to either biotin or the MSD Sulfo-Tag label. Equimolar amounts of these labeled derivatives were mixed with participant serum, added to a streptavidin-functionalized plate, which was subsequently washed before detection of Sulfo-Tag within proximity of the plate surface via electrochemiluminescence, as expressed in relative light units. For samples exhibiting signal exceeding a threshold set during assay development, the specificity of binding was confirmed by repeating the steps above in the presence of 10 μg ml^−1^ of unlabeled 10E8.4/iMab and observing a reduction of signal. Samples in which competition suppressed signal below a threshold defined during assay development were deemed ADA response positive. The magnitude of ADA responses was determined by titering, with response profiles over time also used to define ADAs as either treatment-induced, treatment-boosted, or treatment-independent as previously described^35^. Epitope mapping was performed on a subset of ADA-positive samples, similar to the specificity assessment, but using alternative competitors. CUIMC provided three monoclonal antibodies: iMab, 10E8.4 and 10-1074/3BNC117 CrossMab, and the degree of reduction in signal when using these as competitors was used to define ADA epitope specificity. ADA testing was performed in a Good Clinical Laboratory Practices-compliant environment according to a validated method^36^.

Functional ADA inhibition was measured using a modified version of the TZM.bl HIV-1 neutralization assay as previously described^51^. Briefly, serum samples from select participant timepoints that demonstrated positive ADA binding titers in the MSD assay were serially titrated (primary 1:20 dilution) and tested against HIV-1 Env pseudovirus 3817.v2.c59 in the presence of a constant concentration of 10E8.4/iMab that resulted in approximately 70–80% inhibition of virus infectivity. ADA ID50 titers were measured as the serum dilution that inhibited 50% of 10E8.4/iMab neutralizing activity. Murine anti-idiotype monoclonal antibodies against either wild-type iMab (TaiMed Biologics, Lot 020-JZ-6-168) or 10E8.4 (Syngene International, Lot PRB010089, clone name M30E12B6) were utilized as positive ADA controls.

CD4RO was measured by quantitative flow cytometry based on mean florescent intensity. Whole blood was incubated with (saturation tube) or without (test tube) 20 µg ml^−1^ of 10E8.4/iMab. After washing, staining (with anti-CD45-PerCP, anti-CD3-APC, anti-CD4-FITC Abs and anti-IgG1 Ab-PE) and further incubation, RBC lysis buffer was added, followed by further incubation, washing and fixation. The proportion of the fluorescence intensity of the test solution (without added 10E8.4/iMab) to the fluorescence intensity of the saturated solution (with added 10E8.4/iMab) reflected the CD4RO.

To sequence the HIV-1 envelope gene among participants with active viremia (arm 3 group H), plasma viral RNA was isolated from plasma samples using QIAamp Viral RNA Mini Kit (Qiagen) and used to amplify the envelope gene by the single-genome sequencing method. Viral RNA was reverse transcribed using Superscript III, and the cDNA product was used to amplify the envelope gene by the nested PCR method under the dilution condition that gave the clonal PCR products. Primers used for the nested PCR were as follows: Env5out (5′-TAGAGCCCTGGAAGCATCCAGGAAG-3′) and Env3out (5′- TTGCTACTTGTGATTGCTCCATGT-3′) for the first PCR, and Env5in (5′-CACCTTAGGCATCTCCTATGGCAGGAAGAAG-3′) and Env3in (5′- GTCTCGAGATACTGCTCCCACCC-3′) for the second PCR. Platinum Taq High Fidelity polymerase (Invitrogen) was used to reduce the error rate associated with PCR. The sequence results (Genewiz) were analyzed using Lasergene 17 (DNASTAR).

### Outcomes

The primary study outcome was the rate of signs, symptoms, laboratory abnormalities and solicited AEs within 2 weeks of 10E8.4/iMab infusion and injection in all study arms/groups. Secondary outcomes included the PK profile of 10E8.4/iMab (elimination half-life (t1/2), clearance (CL/F), volume of distribution (Vz/F), AUC and decay curve (serum concentration–time curves)) in all study arms/groups; the decline in plasma HIV-1 RNA level by standard clinical assay after 10E8.4/iMab infusion in PLWH with active viremia; the frequency and levels of induced anti-10E8.4/iMab antibodies in all study groups; and absolute and relative CD4+ and CD8^+^ T cell counts after 10E8.4/iMab infusion. Although bioavailability-adjusted clearance (CL/F) and volume of distribution (Vz/F) are typically estimated by non-compartment approaches, we used population PK modeling to individually estimate each parameter (volume (V_c_), clearance (CL) and bioavailability (F)) as reported in Table 3 due to non-linearity in the PK. AUC was estimated using a non-compartment approach for each participant. A complete list of primary, secondary and exploratory outcomes is provided in Supplementary Information (Protocol).

### PK analysis

#### PK models

All PK analysis considered 10E8.4/iMab serum concentrations measured by ELISA. As the 10E8.4/iMab includes an iMab arm that targets host CD4 receptors, PK was expected to deviate from the standard two-compartment (that is, biphasic) serum kinetics observed in other anti-HIV IgG antibodies^25–27^. To that end, we used a simplifiedTMDD model framework that includes additional kinetics describing antibody binding to host target sites (Fig. 2). Although the fully parameterized model includes CD4 receptor kinetics, we used a Michaelis-Menten approximation^52^, where this process is captured by non-linear clearance via the following ordinary differential equations:

$$\begin{array}{l}\begin{array}{l}\frac{{\rm{d}}D}{{\rm{d}}t}=-{k}_{a}D\,{with}\,D(0)={{Dose}}_{{SC}}\\ \frac{{\rm{d}}C}{{\rm{d}}t}={In}({{Dose}}_{{IV}}/{V}_{c})+F{k}_{a}D/{V}_{c}-\left({k}_{el}+\frac{{V}_{m}}{{K}_{m}+C}+{k}_{12}\right)C+{k}_{21}P/{V}_{c},\\ \frac{{\rm{d}}P}{{\rm{d}}t}={k}_{12}C{V}_{c}-{k}_{21}P.\end{array}\end{array}$$

The central compartment, *C*, describes the serum 10E8.4/iMab concentration over time, *C*(*t*). The model includes a Michaelis-Menten term (*V*_*m*_/(*K*_*m*_ + *C*)), which captures the non-linear clearance from *C* and is parameterized by *V*_*m*_, the maximal non-linear clearance velocity, and *K*_*m*_, the concentration of half-maximal non-linear clearance. The two-compartmental kinetic model describes a linear flow out of *C* with rate ${k}_{{el}}$ and flows between the central and peripheral compartments, P (µg), with flow rates ${k}_{12}$ and ${k}_{21}$, and scaled with central volume, ${V}_{c}$. The model allows for both infusion (${{Dose}}_{{IV}}$ mg dose) and SC (${{Dose}}_{{SC}}$ mg dose) administrations. The *In* function captures infusion dosing based on observed infusion rates for each participant. The depot compartment, *D*, describes the kinetics of 10E8.4/iMab following SC administration as it flows into the central compartment, *C*, at absorption rate, ${k}_{a}$. The SC route bioavailability of the study product is *F*. The serum flow rate parameters were converted to the following standard two-compartment: ${{\rm{V}}}_{{\rm{c}}}$ is the central (plasma) volume, *Cl* is the plasma concentration clearance rate, *Q* is the intercompartmental clearance and ${V}_{p}$ is the volume of the peripheral compartment, using the conversion formula: ${k}_{{el}}={Cl}/{V}_{c},{k}_{12}=Q/{V}_{c},\mathrm{and}\,{k}_{21}=Q/{V}_{p}$. The initial version of the Michaelis-Menten model failed to properly recapitulate kinetics of the PLWoH groups, specifically systematically underpredicting all serum concentration in the high-dose group (Supplementary Fig. 6), indicating an additional dose-dependence beyond what is captured by the standard Michaelis-Menten term. We therefore extended the model to include time-varying ${{\rm{V}}}_{m}$ terms corresponding to the hypothesis that 10E8.4/iMab administration slows CD4 receptor turnover rates. Specifically, the time-dependent Michaelis-Menten model relaxed the stationary assumption of the CD4 receptor turnover using kinetic models that that have been observed for other antibodies deviating from a standard TMDD models^53,54^. The time-dependent ${{\rm{V}}}_{m}$ term was specified as:

$${V}_{m}\left(t\right)={V}_{m,\infty }+\left({V}_{m,0}-{V}_{m,\infty }\right){{\rm{e}}}^{-{v}_{k}t},$$

allowing ${{\rm{V}}}_{m}\left(t\right)$ to decay exponentially over time from a starting value, ${V}_{m,0}$, to a final value, *V*_*m*,∞_, and the change occurs at rate ${v}_{k}$. Comparing the model trajectories between best-fitting models highlights how the time-dependent model better captures the PK for the two high-dose groups. Subsequently, the standard Michaelis-Menten model was outperformed (corrected BICc: 908) compared to the time-dependent model (BICc: 873) for the PLWoH cohort (Supplementary Fig. 6). However, the simpler model without the time-dependent process captured the PLWH PK better (Supplementary Fig. 4).

#### PD model

The PD analysis considered CD4RO percentages measured using flow cytometry. The CD4RO model was specified as

$$\mathrm{CD}4\mathrm{RO}\left(t\right)=\frac{{C\left(t\right)}^{h}}{{C\left(t\right)}^{h}+{{{EC}}_{50}}^{h}},$$

where $C\left(t\right)$ corresponds to the serum concentration of 10E8.4/iMab with two additional parameters, $E{C}_{50}$ and *h*. Here, *EC*_50_ denotes the concentration of the 10E8.4/iMab bispecific antibody that achieves 50% binding of CD4 receptors, whereas the exponent $h$ modulates the rate at which the CD4RO level decays with the concentration of the antibody.

#### Population PK/PD modeling

Population PK/PD models were fitted to observations using non-linear mixed effects models. Models were built and fitted separately to the PLWoH (39 participants) and PLWH (9 participants) cohorts among participants receiving the active product. Each participant in the PLWoH cohort contributed data at 10 post-administration observations and each participant in the PLWH cohort contributed data at 11 post-administration observations. One participant in the PLWH cohort was missing one observation (day 35) during the study. For a given model, population-level parameters were represented as fixed effects and individual-level variation of PK parameters around their population means were described by random effects. The standard deviations of random effects are generically denoted by $\omega$, with a subscript identifying the PK parameter to which it applies. The residual error model was specified to capture statistical properties of the outcome measure. Models describing PK were fit to 10E8.4/iMab serum concentrations assuming a normal distribution for the error model with concentrations below the LLoQ (0.2 µg ml^−1^) treated as left-censored observations. Models including a PD component were also fitted to CD4RO observations assuming a logit-normal distribution (bounded between 0 and 100%) for the error model. For both of these outcomes, the conditional variance of the error term, given the random effects, was assumed to be proportional to the conditional expectation of the concentration. Subject-specific model parameters were assumed to follow log-normal distributions, except for bioavailability assumed to be logit-normal (bound between 0% and 100%). Population parameters (mean and variance of random parameters), fixed effects, and parameters of the error term variance were estimated using the method of maximum likelihood, implemented using the stochastic approximation expectation maximization algorithm using MonolixSuite (v2024R1, Lixoft; Simulations Plus)^55^. Reported standard errors were estimated using a linearization approximation method implemented by the software. For the PLWoH groups, models were initially fit to the IV groups only (SC groups excluded); once a model was established, the SC groups were included in the model fitting to determine the absorption and bioavailability. Initial PK models included random effects specified for all two-compartment population PK parameters (*Cl*, *V*_*c*_*, V*_*p*_ and *Q*) and the velocity term, *V*_*m*_. The inclusion of random effects (inter-subject variability) was assessed during model building by evaluating whether the relative standard error (standard deviation/mean) was below 50%. Ultimately, random effects were only included on the *Cl* and *V*_*c*_ terms. For the PD models, a random effect was initially included on the *EC*_50_ parameter and assessed similarly as the PK parameter random effects. Models were otherwise compared using BICc^56^ with the lowest value being used to determine the final model structure. For both PLWoH and PLWH cohorts, the *K*_*m*_ parameter was difficult to estimate and was fixed to the LLoQ (0.2 µg ml^−1^) after a sensitivity analysis was performed showing that the other parameter estimates were insensitive to a range of potential fixed *K*_*m*_ between 0.2 and 10^−6^ µg ml^−1^, suggesting the true *K*_*m*_ likely falls below the range of the observed data. Baseline weight and CD4 cell count (percentages) were considered as covariates. For the time-dependent Michaelis-Menten in the PLWoH cohort, assessment of covariates failed as the model fitting algorithm failed to converge with additional specification. For the PLWH cohort, the stepwise covariate modeling algorithm was implemented in Monolix. The stepwise covariate modeling performs iterative forward followed by backward selection to determine whether any given candidate covariate adjustment should be included in the model. This evaluation is based on a two-sided likelihood ratio test with a significance level (that is, type-I error rate) alpha set at 10%. During each step of the forward selection process, the significance of the parameter describing each covariate adjustment is evaluated using the test. At the conclusion of the step, only one covariate is added to the model; if multiple coefficients meet the inclusion criteria, the model is selected via the highest log-likelihood. This process repeats until no significant covariate adjustment is added to the model. The model selected via forward selection is next subjected to backward selection to eliminate unnecessary (that is, nonsignificant) covariate adjustment by following a process similar to that used by forward selection. Potential candidate covariate-adjusted models were compared via BICc (Supplementary Table 4). The fitting of the PD model was done using a sequential approach. After the PK model was fitted to serum concentrations, the full PK/PD model was then fitted to the serum concentration and CD4RO percentage simultaneously, estimating only the PD parameters (fixed and random effects) while keeping the population PK parameters fixed.

#### Model simulations

Population trajectories were simulated from the fitted models with both variability and uncertainty. To generate prediction intervals, sampling was performed by the Simulx software^55^: Bootstrap sampling with 10,000 replicates were sampled from each population parameter set allowing variation via model-estimated random effects. A curve was then simulated for each replicate. For each timepoint, a 90% prediction interval was then estimated using the 5th and 95th of the simulated values. This analysis was repeated for each dose group using the mean observed dose.

#### Elimination half-life

The asymptotic, maximal half-life represents the half-life of the TMDD model either when there are no available CD4 receptors to occupy (that is, the model reduces to a two-compartment model), or when antibody concentrations approach infinity. Subsequently, the maximal half-life can be computed from the two-compartment parameters as ${{\rm{t}}}_{1/2}=\log \left(2\right)/\beta$/2 where $\beta =-{({k}_{12}+k}_{21}{+k}_{{el}})-\sqrt{{{({k}_{12}+k}_{21}{+k}_{{el}})}^{2}-4{k}_{21}{k}_{{el}}}$. We also computed an approximate half-life representing a transitive phase when CD4 receptors were fully occupied and product clearance was approximately linear. This approximate half-life is dose and regimen dependent and represents the slowest product clearance phase for a given dose and regimen (for example, administration route). To calculate these half-lives, we determined the shallowest clearance phase by performing a series of regressions on simulated concentration–time curve based on the mean population PK parameters and median administered dose for each of the two groups. The simulated data used in the regression model started on day 3 to remove the distribution phase and ended on day 28, corresponding to the last observation study time point with positive serum concentrations. In each model, we regressed log-transformed concentrations on time (days). A model was fit for every possible combination of the first and last study day for the simulated concentration data (between days 3 and 28) with a minimum of a 7-day window. The fitted regression model with the slowest clearance was assumed to capture the slowest linear phase. The estimated slope of that model was used to calculate the approximate half-life (as *log*(0.5)/slope). All reported approximate half-life estimates came from with models with R-squared >98%.

#### VL analysis

HIV VL was measured over time in the three participants enrolled in group H. Viral reduction was calculated as the log_10_-difference in VL between baseline (time at which antibody infusion started) and nadir (minimum). Antiviral activity for plasma VL under combination ART has been previously well-described and shown to follow a biphasic decay over the first several weeks that follow treatment^40,41,44,57^. Under the assumption that ART was fully effective at interrupting viral replication, the initial (faster) phase of VL decline is determined by the death rate of actively infected peripheral target cells: peripheral CD4^+^ T cells expressing HIV-targeted co-receptors^40,41,57^. The second (slower) phase of VL decline is thought to be determined by the death rate of a broader class of long-lived, infected target cell types. The VL at time *t* following activation of drug therapy is then given by^41^:

$$V\left(t\right)={V}_{0}\cdot \left[{A}_{1}{e}^{-\delta t}+{A}_{2}{e}^{-{\mu }_{M}t}+\left(1-{A}_{1}-{A}_{2}\right){e}^{-{ct}}\right],$$

where

$${A}_{1}=\frac{K}{c-\delta },$$

and

$${A}_{2}=\frac{c-K}{c-{\mu }_{M}}.$$

Here, ${V}_{0}$ stands for VL at time *t* = 0, the time of drug activation (that is, the time at which antiviral activity begins), which may follow a short lag period (1–2 days) following drug initiation. The parameter $\delta$ denotes the death rate of peripheral infected target cells (per day); ${\mu }_{M}$ denotes the death rate of long-lasting infected cells (per day); $c$ denotes the clearance rate of free plasma virus (per day); and $K$ is a composite parameter describing the interaction between the virus and susceptible cells based on the law of mass action. The first phase half-life is calculated as log(2)/$\delta$ and the second-phase half-life is calculated as log(2)/${\mu }_{M}$. The clearance rate of free plasma virus has been independently estimated as *c* = 23 per day^44^.

As there was limited serial sampling, we applied the ART antiviral model to 10E8.4/iMab assuming equivalent effects. To that end, we fixed key parameter values to those estimated from clinical populations treated with ART^43,44^. For the initial VL decline phase, we assumed that 10E8.4/iMab initially fully interrupted the viral replication process and fixed the first phase half-life to log(2)/ $\delta$ = 0.69 days; and for the second phase, we assumed a half-life of 25 days. The only model parameter that we estimated from the data was the *K* parameter for each participant (a determinant of the inflection point of the biphasic curve), as estimates for this parameter were not readily available from previous studies. The starting value for *K* was set to 15. We also set a 2-day lag period that was similar to lag periods observed in the ART studies. The model therefore started at day 2 with the measurement at day 2 used as ${V}_{0}$. Models were fit via non-linear least squares using the Golub-Pereyra algorithm implemented in R (nls function).

#### Drug exposure (AUC)

Drug exposure was quantified as the area under the concentration–time curve (AUC), calculated using the linear/log trapezoidal method. Linear trapezoids were applied for intervals before observed maximum concentration, and log-linear trapezoids for intervals after maximum concentration. Total AUC for each participant was obtained by summing all partial intervals.

### Reporting summary

Further information on research design is available in the Nature Portfolio Reporting Summary linked to this article.

## Online content

Any methods, additional references, Nature Portfolio reporting summaries, source data, extended data, supplementary information, acknowledgements, peer review information; details of author contributions and competing interests; and statements of data and code availability are available at 10.1038/s41591-026-04472-w.

## Supplementary information

Supplementary Information Supplementary Figures 1–6, Supplementary Tables 1–4, CONSORT checklist.

Reporting Summary

## Figures and Tables

**Extended Data Table 1:**
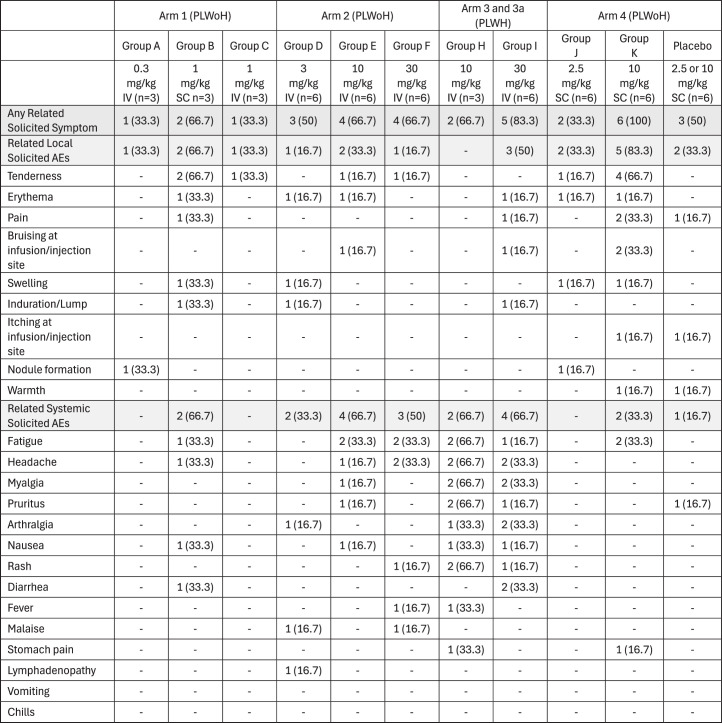
Related solicited adverse events (AEs) occurring within two weeks of 10E8.4/iMab administration in all study arms/groups

**Extended Data Table 2:**
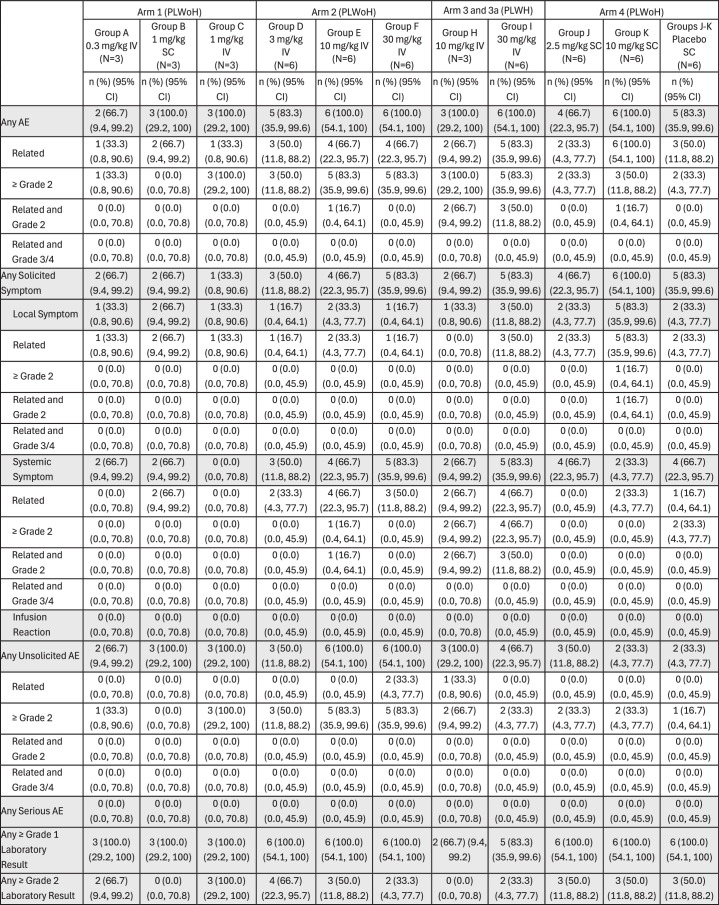
Solicited adverse events (AEs) and unsolicited AEs (signs, symptoms and laboratory abnormalities) occurring during study follow-up (after 10E8.4/iMab administration) in all study arms/groups

**Extended Data Table 3:**
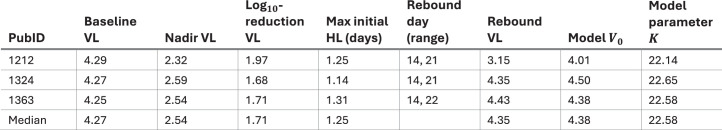
Summary of plasma viral load (VL) results for each participant in Group H (10 mg kg^−1^)

Nadir (minimum) plasma VL was measured at day 14 for all participants. Log_**10**_ fold-reduction in VL was calculated between baseline and nadir. Maximum (slowest) half-life was calculated assuming a linear reduction between VL measurements on days 2 and 7 for comparison to combination antiretroviral treatment (cART) first-phase half-life of 0.67 days. Rebound time (range) is interval censored between nadir day and the following observation time point. For more details on model parameters (***V***_**0**_ and ***K***), see Methods. Viral load measurements are log_**10**_ concentration (copies/mL).

**Extended Data Table 4:**
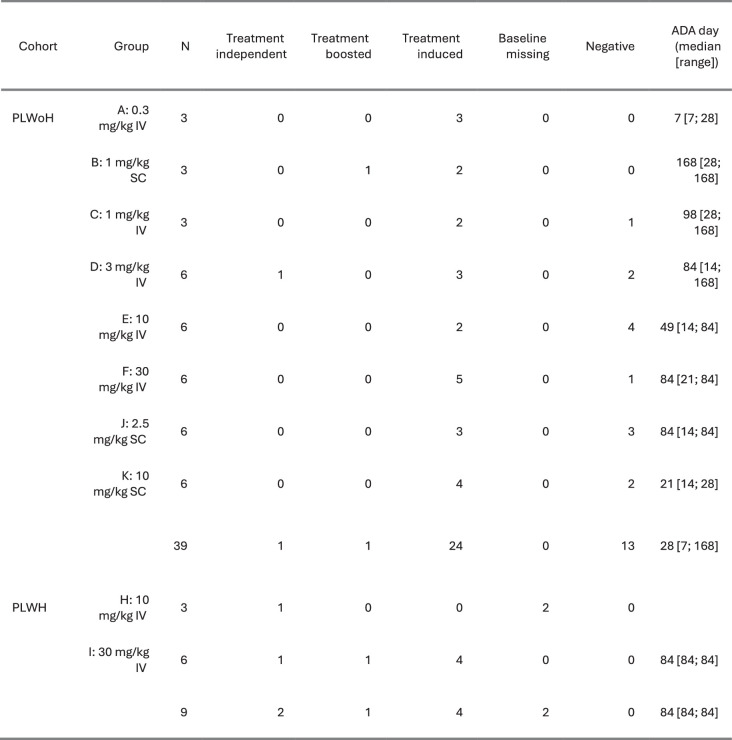
Anti-drug antibody (ADA) status counts by study group among PLWoH and PLWH

The ADA day column represents the study day at which ADA were detected among participants with treatment-related ADA responses (treatment-boosted or treatment-induced responses). Responses for two participants with missing baseline ADA assays could not be categorized; both participants had a positive ADA titer during the study.

## Data Availability

The full analyzable data set (all participant-level data collected in the study, including safety and immunogenicity data) and all versions of the protocol used in the trial will be available indefinitely through the Vivli platform at 10.25934/PR00012686.

## Code availability

All R code required to reproduce the results are available at https://github.com/HyrienLab/ABA0101Manuscript/. The code supporting the shiny application is available at https://github.com/FredHutch/Sim10e8imab.
