# Supplementary material for: Bispecific 10E8.4/iMab broadly neutralizing antibody in people with or without HIV-1: a partially randomized phase 1 trial
Source: Nat Med. 2026 Jul 7;32(7):2533–45. doi: 10.1038/s41591-026-04472-w (PMC13375543; doi:10.1038/s41591-026-04472-w)
Supplement: Supplementary file 1 — Supplementary Figures 1–6, Supplementary Tables 1–4, CONSORT checklist. [file 41591_2026_4472_MOESM1_ESM.pdf]

nature medicine

Supplementary information https://doi.org/10.1038/s41591-026-04472-w

# Bispecific 10E8.4/iMab broadly neutralizing antibody in people with or without HIV-1: a partially randomized phase 1 trial

In the format provided by the authors and unedited

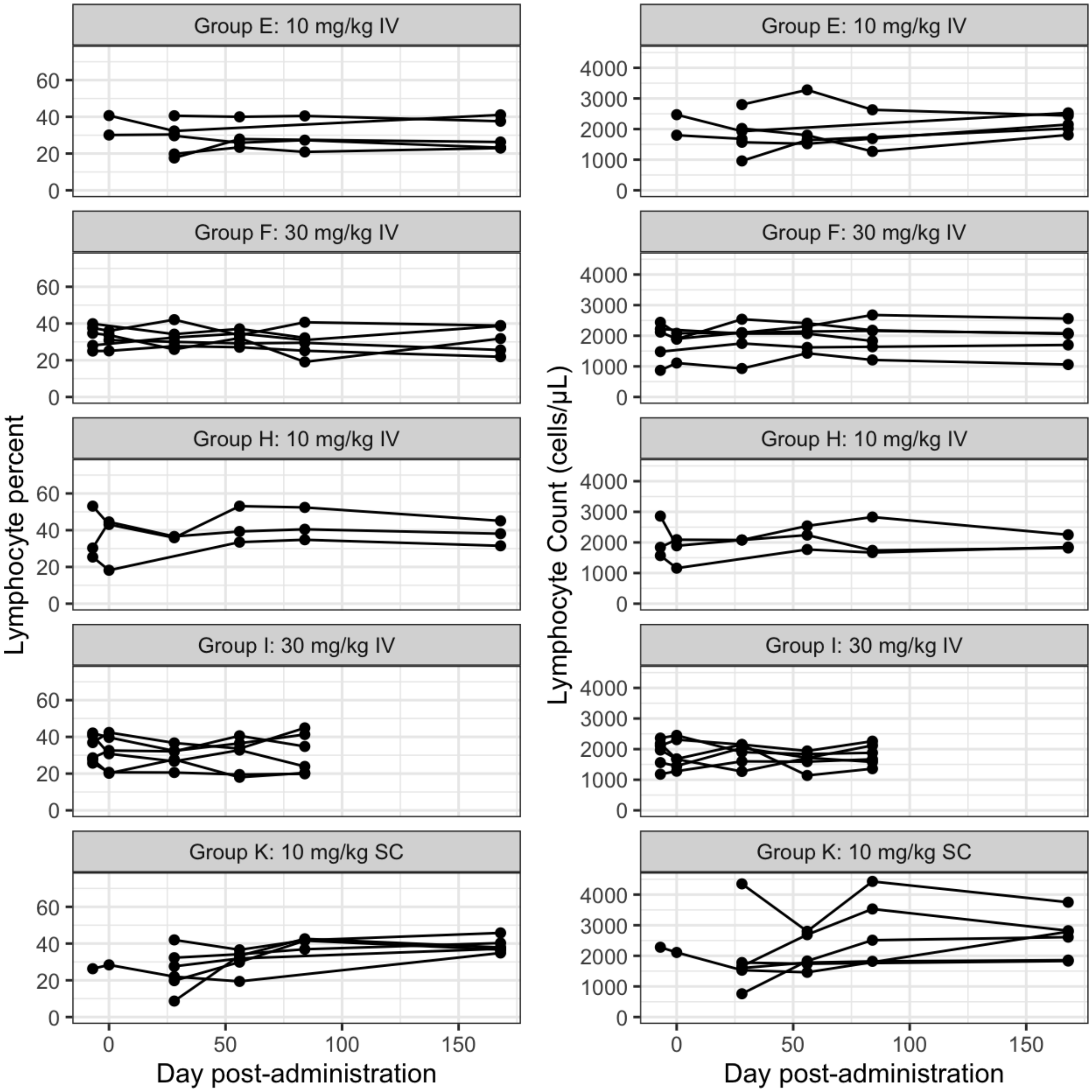


**Supplementary Fig. 1. Lymphocyte cell counts and percentages of total white blood cells over time in PLWoH (Groups E, F, and K) and PLWH (Groups H and I).**

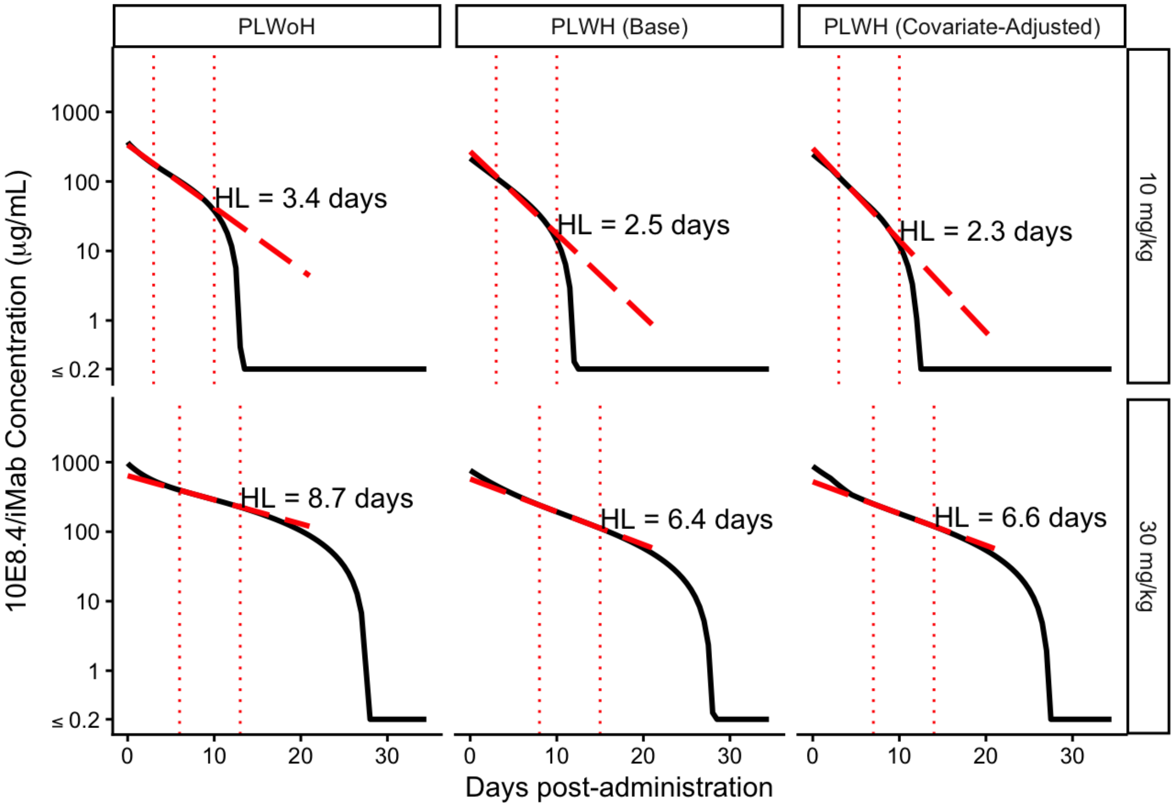


**Supplementary Fig. 2. Estimation of approximate half-life (HL) by finding slowest linear phase (dashed red line) overlaid on serum concentration curve.** Population serum concentration curves computed based on the population PK parameters. Approximate HL computed for high dose groups (10 mg/kg and 30 mg/kg) for each cohort (PLWH and PLWoH). Vertical dotted lines indicate range (minimum 1 week) of data where longest HL estimated.

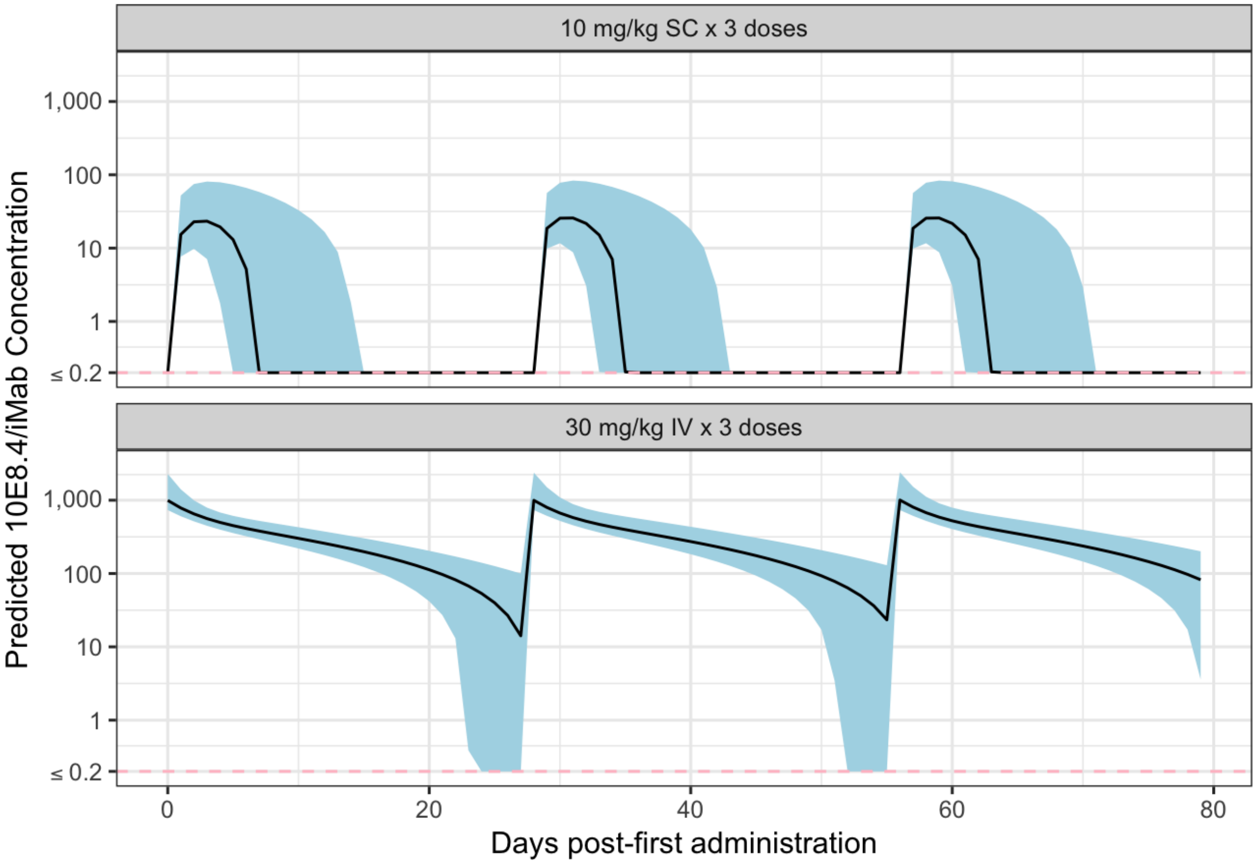


**Supplementary Fig. 3. Simulated 10E8.4/iMab concentrations in PLWoH over time (days relative to study administration) from two dosing scenarios: 10 mg/kg subcutaneous (SC) and 30 mg/kg intravenous (IV).** In both scenarios, 3 doses were administered 28 days apart. The solid line is the simulated median concentration, and the blue shaded area is the 90% prediction interval. The red dashed line represents the lower limit of quantification.

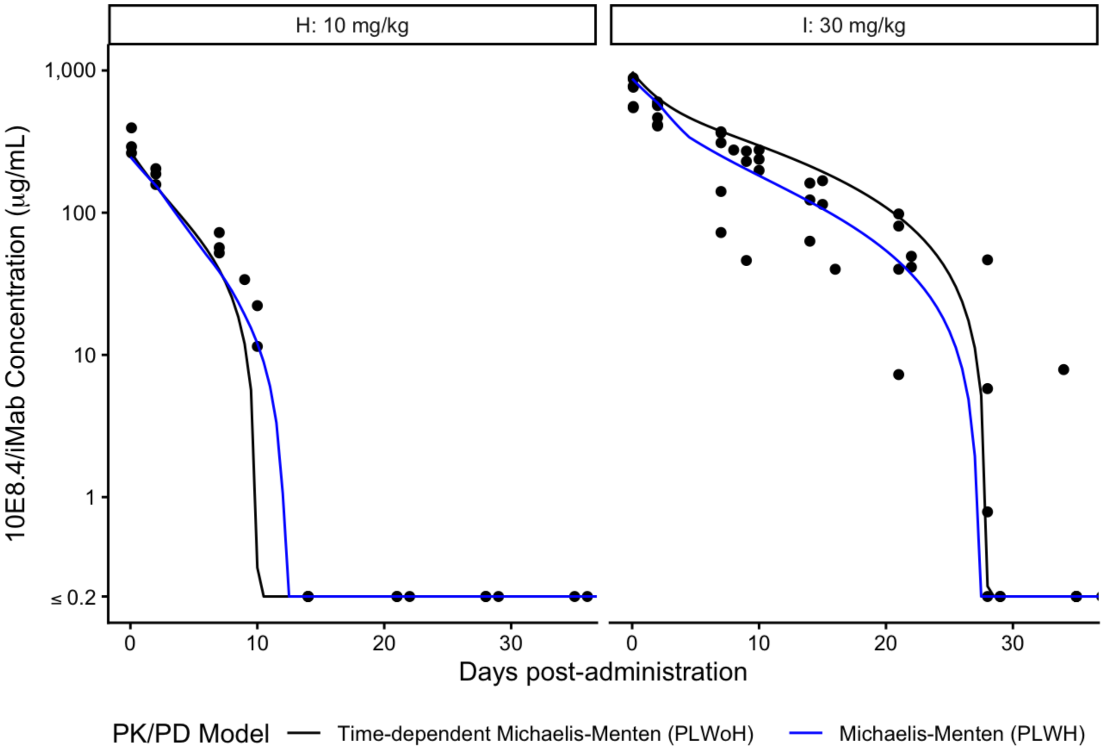


**Supplementary Fig. 4. Observed 10E8.4/iMab individual concentrations with population-predicted PK (group mean dose; lines, colored by model-type) among PLWH (Groups H and I).** The lines depict the predicted population PK using the time-dependent Michelis-Menton PK/PD model trained to either the PLWoH (see also Supp Fig 1) or re-trained to PLWH (see Table 2 for parameters). When the model was fit to PLWH, it reduced to a simpler Michaelis-Menten model. The model for PLWH was covariate-adjusted.

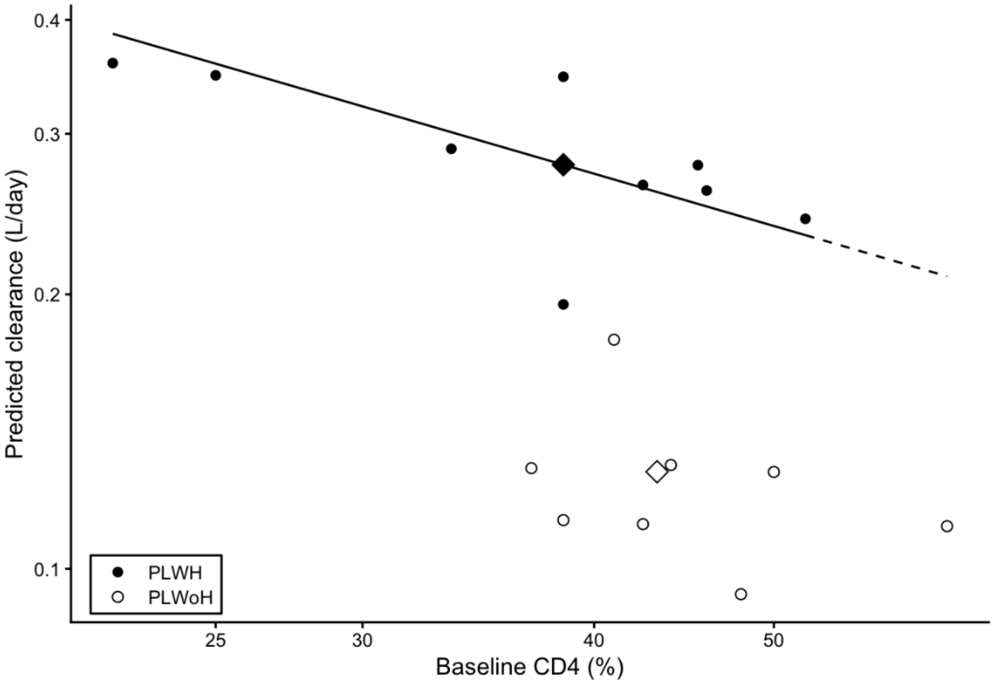


**Supplementary Fig. 5. Estimated relationship between model-predicted central compartment clearance (L/day) and observed baseline CD4 percent among PLWH (solid points) and PLWoH (Groups E and F, open points).** The corresponding diamonds indicate the median baseline CD4% and the population estimated clearance values from the model. The line depicts the covariate relationship estimated by the covariate-adjusted PK model among participants with HIV, with the solid portion of the line representing the baseline CD4 percent range observed in this cohort and its dashed portion representing extrapolated predictions for the baseline CD4 percent range observed in the PLWoH.

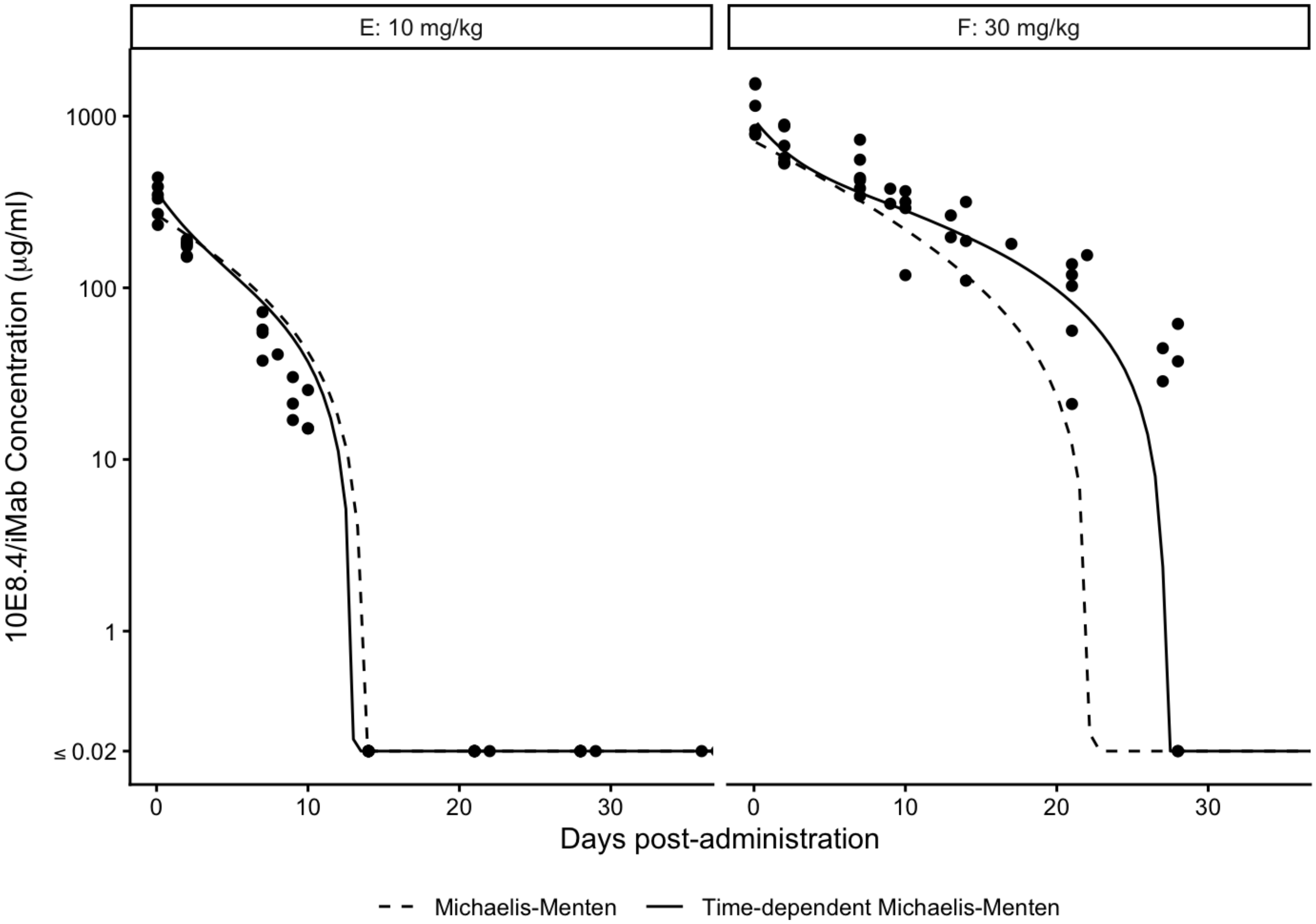


**Supplementary Fig. 6. Observed 10E8.4/iMab individual concentrations with population-predicted PK (group mean dose; lines, line type by model-type) for the high dose IV groups among PLWoH (Groups E and F).** The time-dependent Michaelis-Menten was selected as the final model for this cohort both based on model information criteria and the ability of the model to capture the high dose group more accurately.

| COHORT | GROUP | N | NCA N | AUC | DOSE-ADJUSTED AUC |
|---|---|---|---|---|---|
| PLWoH | Group A: 0.3 mg/kg IV | 3 | 3 | 10.33 [9.55; 11.14] | 0.55 [0.39; 0.56] |
| | Group B: 1 mg/kg SC | 3 | 0 | | |
| | Group C: 1 mg/kg IV | 3 | 3 | 16.52 [14.78; 17.40] | 0.31 [0.22; 0.34] |
| | Group D: 3 mg/kg IV | 6 | 6 | 132.74 [97.93; 187.18] | 0.55 [0.40; 0.71] |
| | Group E: 10 mg/kg IV | 6 | 6 | 1155.20 [857.58; 1253.93] | 1.28 [1.02; 1.62] |
| | Group F: 30 mg/kg IV | 6 | 6 | 7666.68 [5532.41; 11177.25] | 3.58 [2.17; 4.84] |
| | Group J: 2.5 mg/kg SC | 6 | 3 | 12.48 [9.36; 13.85] | 0.07 [0.06; 0.09] |
| | Group K: 10 mg/kg SC | 6 | 6 | 51.50 [25.67; 106.23] | 0.09 [0.04; 0.19] |
| PLWH | Group H: 10 mg/kg IV | 3 | 3 | 1218.56 [1001.38; 1247.97] | 1.79 [1.17; 1.89] |
| | Group I: 30 mg/kg IV | 6 | 6 | 5111.54 [2486.14; 6670.24] | 2.08 [1.43; 2.51] |

**Supplementary Table 1. Area under the curve (AUC, median [range]) drug exposure estimates for serum concentrations of 10E8.4/iMab pharmacokinetics by group and cohort.** AUC values were computed using non-compartment analysis (NCA) based on the observed concentrations that were above the lower limit of quantification (> LLoQ, 0.2 $\mu$g/mL). If a participant did not have multiple observed concentrations above detection, they were not included in the computation (NCA N). Dose-adjusted AUC scaled by the amount of antibody administered.

**Participant 1324**

| virus sequence (n) | iMab-associated resistance C3 (335-344) | V5 (458-467) | 10E8.4-associated resistance MPER (671-683) |
|---|---|---|---|
| JRCSF-WT | RAQWNNTLKQ | GGKNESEIEI | NWFGITKWLWYIK |
| 1324 day 0 (5) | RTKWNETLHK | GGNNESGVET | NWFDITNWLWYIK |
| 1324 day 0 (1) | RTKWNETLHK | GGNNESEAET | NWFDITNWLWYIK |
| | | | |
| 1324 day 14 (1) | RTKWNETLHK | GGNNESEMET | NWFDITNWLWYIK |
| | | | |
| 1324 day 21 (2) | RTKWNETLHK | GGNNESEMET | NWFDITNWLWYIK |
| 1324 day 21 (2) | RTKWNETLQK | GGNNESEVET | NWFDITNWLWYIK |
| | | | |
| Summary: | No change at N position.<br>H343Q appeared. | No change at S position.<br>G464E increased.<br>V465M appeared. | No change at WF positions. |

**Participant 1363**

| virus sequence (n) | iMab-associated resistance C3 (335-344) | V5 (458-467) | 10E8.4-associated resistance MPER (671-683) |
|---|---|---|---|
| JRCSF-WT | RAQWNNTLKQ | GGKN-ESEIEI | NWFGITKWLWYIK |
| 1363 day 0 (4) | GESWNKALEK | GGHNGTSPNET | NWFDISNWLWYIK |
| | | | |
| 1363 day 21 (4) | GESWNKALEK | GGHNGTSPNET | NWFDISNWLWYIK |
| | | | |
| Summary: | No change at N positions. | No change at S positions. | No change at WF positions. |

**Participant 1212**

| virus sequence (n) | iMab-associated resistance C3 (335-344) | V5 (458-467) | 10E8.4-associated resistance MPER (671-683) |
|---|---|---|---|
| JRCSF-WT | RAQWNNTLKQ | GGKNESE-IEI | NWFGITKWLWYIK |
| 1212 day 0 (1) | GTAWNNTLKQ (1) | GGNNQTNKTET (1) | NWFDISNWLWYIK (1) |
| | | | |
| 1212 day 21 (2) | GTAWNNTLKQ (2) | GGNNQTNKTET (1) | NWFDISNWLWYIK (1) |
| | | GGNNQTN--ET (1) | NWFGISNWLWYIK (1) |
| | | | |
| 1212 day 28 (1) | GTAWNNTLKQ (1) | GGNNQTNKTET (1) | NWFDISNWLWYIK (1) |
| | | | |
| Summary: | No change at N positions. | No change at S positions. | Mo change at WF positions.<br>D674G appeared. |

**Supplementary Table 2. Sequencing of HIV-1 env gene in rebound plasma virus after 10 mg/kg of 10E8.4/iMab IV administration (Arm 3 Group H).** Amino acid changes at the position noted in red in JRCSF-WT are associated with resistance.

| Participant ID (Group) | Timepoint | Serum ADA ID50 Titer in TZM.bl Cells | Participant ID (Group) | Timepoint | Serum ADA ID50 Titer in TZM.bl Cells | Participant ID (Group) | Timepoint | Serum ADA ID50 Titer in TZM.bl Cells |
|---|---|---|---|---|---|---|---|---|
| 0388 (A) | V01 | <20 | 0814 (D) | V05 | <20 | 0250 (K) | V07 | <20 |
| | V03 | <20 | | V06 | 25 | | V11 | 21 |
| | V05 | <20 | | V07 | 22 | 0597 (K) | V03 | <20 |
| | V06 | <20 | | V11 | <20 | | V05 | <20 |
| | V07 | <20 | 0558 (D) | V05 | 34 | | V06 | <20 |
| | V10 | 31 | | V06 | 26 | | V07 | <20 |
| | V11 | 22 | | V07 | 29 | | V11 | <20 |
| 0392 (A) | V01 | <20 | | V10 | <20 | 1737 (K) | V06 | <20 |
| | V03 | <20 | 1636 (D) | V01 | 47 | | V07 | <20 |
| | V05 | <20 | | V05 | 49 | | V10 | 29 |
| | V07 | <20 | | V06 | 54 | | V11 | <20 |
| | V10 | <20 | | V07 | 63 | 1792 (K) | V05 | <20 |
| 0281 (A) | V01 | <20 | | V10 | 81 | | V06 | <20 |
| | V07 | <20 | 1995 (E) | V10 | <20 | | V07 | <20 |
| | V10 | <20 | 1887 (E) | V05 | <20 | | V10 | 54 |
| | V11 | <20 | | V06 | <20 | | V11 | 46 |
| 1033 (B) | V01 | <20 | | V07 | <20 | 0129 (K) | V06 | 33 |
| | V07 | <20 | | V10 | 33 | | V07 | 37 |
| | V10 | <20 | | V11 | 37 | | V10 | 54 |
| | V11 | <20 | 1662 (F) | V05 | <20 | | V11 | 125 |
| 0889 (B) | V01 | <20 | | V10 | <20 | 1324 (H) | V06 | <20 |
| | V07 | <20 | | V11 | <20 | | V07 | <20 |
| | V10 | <20 | 1578 (F) | V05 | <20 | | V10 | <20 |
| | V11 | <20 | | V10 | <20 | | V11 | <20 |
| 1085 (B) | V01 | <20 | | V11 | <20 | 1363 (H) | V05 | <20 |
| | V06 | <20 | 1713 (F) | V05 | <20 | | V06 | <20 |
| | V07 | <20 | | V10 | <20 | | V07 | <20 |
| | V10 | <20 | | V11 | <20 | | V10 | <20 |
| | V11 | <20 | 0497 (F) | V05 | <20 | 1212 (H) | V01 | <20 |
| 0024 (C) | V01 | <20 | | V06 | <20 | | V03 | <20 |
| | V07 | <20 | | V07 | <20 | | V05 | <20 |
| | V10 | <20 | | V10 | <20 | | V06 | <20 |
| | V11 | <20 | | V11 | 30 | | V07 | <20 |
| 0049 (C) | V01 | <20 | 0983 (F) | V05 | <20 | | V10 | <20 |
| | V07 | <20 | | V10 | <20 | 1409 (I) | V05 | <20 |
| | V10 | <20 | | V11 | 24 | | V06 | <20 |
| | V11 | <20 | 0627 (J) | V01 | <20 | | V10 | <20 |
| 0101 (C) | V01 | <20 | | V03 | <20 | 1450 (I) | V01 | <20 |
| | V07 | <20 | | V05 | <20 | | V05 | <20 |
| | V10 | <20 | | V06 | <20 | | V07 | <20 |
| 0967 (D) | V05 | <20 | | V07 | <20 | | V10 | <20 |
| | V06 | <20 | | V10 | <20 | 1459 (I) | V05 | <20 |
| | V07 | <20 | | V11 | <20 | | V07 | <20 |
| | V10 | <20 | 0818 (J) | V10 | <20 | | V10 | <20 |
| | V11 | <20 | | V11 | <20 | 0906 (I) | V05 | <20 |
| 0786 (D) | V01 | <20 | 0853 (J) | V05 | <20 | | V10 | <20 |
| | V10 | <20 | | V06 | <20 | 0685 (I) | V05 | <20 |
| 0295 (D) | V05 | <20 | | V07 | <20 | | V10 | <20 |
| | V06 | <20 | | V10 | <20 | 0987 (I) | V01 | <20 |
| | V07 | <20 | 0693 (J) | V10 | <20 | | V06 | <20 |
| | V10 | <20 | | V11 | <20 | | V10 | <20 |

**Supplementary Table 3. ADA measurement as a function of reduced 10E8.4/iMab neutralizing activity.** The baseline sample (V01) from participant 1636 showed functional inhibitory activity, suggesting background inhibition that was not related to receipt of 10E8.4/iMab. Visit 01 (V01) corresponds to day 0, V03 to day 7, V05 to day 14 (±1 day), V06 to day 21 (±1 day), V07 to day 28 (±1 day), V10 to day 84 (±7 days), and V11 to day 168 (±14 days).

| Model iteration | Covariate-adjustment | BICc |
|---|---|---|
| 1 | None | 584 |
| 2 | Vc:CD4 | 584 |
| 3 | Vc:WT | 578 |
| 4 | Cl:CD4 | 582 |
| 5 | Cl:WT | 584 |
| 6 | Vm:CD4 | 587 |
| 7 | Vm:WT | 588 |
| 8 | Vc:CD4, V:WT | 579 |
| **9** | **Vc:WT, Cl:CD4** | **576** |
| 10 | Vc:WT, Cl:WT | 580 |
| 11 | Vc:WT, Vm:CD4 | 581 |
| 12 | Vc:WT, Vm:WT | 582 |
| 13 | Vc:CD4, Vc:WT, Cl:CD4 | 577 |
| 14 | Vc:WT, Cl:CD4, Cl:WT | 577 |
| 15 | Vc:WT, Vm:CD4, Cl:CD4 | 579 |
| 16 | Vc:WT, Vm:WT, Cl:CD4 | 580 |

**Supplementary Table 4. Information criteria (corrected Bayesian information criteria, BICc) estimated for the PK models during covariate model building using the stepwise covariate modeling algorithm in the PLWH cohort.** Covariate-adjustments were implemented using a log-linear function with all covariates log-transformed and centered on their median. The covariates tested were baseline weight (WT, kg) and CD4-levels (%). Adjustments included in the given model are denoted by parameter:covariate. The final model was bolded and highlighted gray (row 9).

| Section/topic | No | CONSORT 2025 checklist item description | Reported on page no. |
|---|---|---|---|
| **Title and abstract** | | | |
| Title and structured abstract | 1a | Identification as a randomised trial | 1 |
| | 1b | Structured summary of the trial design, methods, results, and conclusions | 2 |
| **Open science** | | | |
| Trial registration | 2 | Name of trial registry, identifying number (with URL) and date of registration | 21 |
| Protocol and statistical analysis plan | 3 | Where the trial protocol and statistical analysis plan can be accessed | 30 |
| Data sharing | 4 | Where and how the individual de-identified participant data (including data dictionary), statistical code and any other materials can be accessed | 34 |
| Funding and conflicts of interest | 5a | Sources of funding and other support (eg, supply of drugs), and role of funders in the design, conduct, analysis and reporting of the trial | 12 |
| | 5b | Financial and other conflicts of interest of the manuscript authors | 12 |
| **Introduction** | | | |
| Background and rationale | 6 | Scientific background and rationale | 3 |
| Objectives | 7 | Specific objectives related to benefits and harms | 3 |
| **Methods** | | | |
| Patient and public involvement | 8 | Details of patient or public involvement in the design, conduct and reporting of the trial | 21 |
| Trial design | 9 | Description of trial design including type of trial (eg, parallel group, crossover), allocation ratio, and framework (eg, superiority, equivalence, non-inferiority, exploratory) | 21 |
| Changes to trial protocol | 10 | Important changes to the trial after it commenced including any outcomes or analyses that were not prespecified, with reason | 25-26 |
| Trial setting | 11 | Settings (eg, community, hospital) and locations (eg, countries, sites) where the trial was conducted | 21 |
| Eligibility criteria | 12a | Eligibility criteria for participants | 21-25 |
| | 12b | If applicable, eligibility criteria for sites and for individuals delivering the interventions (eg, surgeons, physiotherapists) | NA |
| Intervention and comparator | 13 | Intervention and comparator with sufficient details to allow replication. If relevant, where additional materials describing the intervention and comparator (eg, intervention manual) can be accessed | 27-30 |
| Outcomes | 14 | Prespecified primary and secondary outcomes, including the specific measurement variable (eg, systolic blood pressure), analysis metric (eg, change from baseline, final value, time to event), method of aggregation (eg, median, proportion), and time point for each outcome | 30 |
| Harms | 15 | How harms were defined and assessed (eg, systematically, non-systematically) | 27-28 |
| Sample size | 16a | How sample size was determined, including all assumptions supporting the sample size calculation | 26 |
| | 16b | Explanation of any interim analyses and stopping guidelines | NA |
| Randomisation: | | | |
| Sequence generation | 17a | Who generated the random allocation sequence and the method used | 27 |
| | 17b | Type of randomisation and details of any restriction (eg, stratification, blocking and block size) | 27 |

| | | | Reported on page no. |
|---|---|---|---|
| Allocation concealment mechanism | 18 | Mechanism used to implement the random allocation sequence (eg, central computer/telephone; sequentially numbered, opaque, sealed containers), describing any steps to conceal the sequence until interventions were assigned | 27 |
| Implementation | 19 | Whether the personnel who enrolled and those who assigned participants to the interventions had access to the random allocation sequence | 27 |
| Blinding | 20a | Who was blinded after assignment to interventions (eg, participants, care providers, outcome assessors, data analysts) | 27 |
| | 20b | If blinded, how blinding was achieved and description of the similarity of interventions | 27 |
| Statistical methods | 21a | Statistical methods used to compare groups for primary and secondary outcomes, including harms | 26-27 |
| | 21b | Definition of who is included in each analysis (eg, all randomised participants), and in which group | 26 |
| | 21c | How missing data were handled in the analysis | 31 |
| | 21d | Methods for any additional analyses (eg, subgroup and sensitivity analyses), distinguishing prespecified from post hoc | NA |
| **Results** | | | |
| Participant flow, including flow diagram | 22a | For each group, the numbers of participants who were randomly assigned, received intended intervention, and were analysed for the primary outcome | Figure 1 |
| | 22b | For each group, losses and exclusions after randomisation, together with reasons | Figure 1 |
| Recruitment | 23a | Dates defining the periods of recruitment and follow-up for outcomes of benefits and harms | 4 |
| | 23b | If relevant, why the trial ended or was stopped | NA |
| Intervention and comparator delivery | 24a | Intervention and comparator as they were actually administered (eg, where appropriate, who delivered the intervention/comparator, how participants adhered, whether they were delivered as intended (fidelity)) | 3-5 |
| | 24b | Concomitant care received during the trial for each group | 4-5 |
| Baseline data | 25 | A table showing baseline demographic and clinical characteristics for each group | Table 1 |
| Numbers analysed, outcomes and estimation | 26 | For each primary and secondary outcome, by group:<br>● the number of participants included in the analysis<br>● the number of participants with available data at the outcome time point<br>● result for each group, and the estimated effect size and its precision (such as 95% confidence interval)<br>● for binary outcomes, presentation of both absolute and relative effect size | 5-9 |
| Harms | 27 | All harms or unintended events in each group | 5-6 |
| Ancillary analyses | 28 | Any other analyses performed, including subgroup and sensitivity analyses, distinguishing pre-specified from post hoc | NA |
| **Discussion** | | | |
| Interpretation | 29 | Interpretation consistent with results, balancing benefits and harms, and considering other relevant evidence | 9-12 |
| Limitations | 30 | Trial limitations, addressing sources of potential bias, imprecision, generalisability, and, if relevant, multiplicity of analyses | 9-12 |

Citation: Hopewell S, Chan AW, Collins GS, Hróbjartsson A, Moher D, Schulz KF, et al. CONSORT 2025 Statement: updated guideline for reporting randomised trials. BMJ. 2025; 388:e081123. https://dx.doi.org/10.1136/bmj-2024-081123
© 2025 Hopewell et al. This is an Open Access article distributed under the terms of the Creative Commons Attribution License (https://creativecommons.org/licenses/by/4.0/), which permits unrestricted use, distribution, and reproduction in any medium, provided the original work is properly cited.

*We strongly recommend reading this statement in conjunction with the CONSORT 2025 Explanation and Elaboration and/or the CONSORT 2025 Expanded Checklist for important clarifications on all the items. We also recommend reading relevant CONSORT extensions. See www.consort-spirit.org.
