# Supplementary material for: Bispecific 10E8.4/iMab broadly neutralizing antibody in people with or without HIV-1: a partially randomized phase 1 trial
Source: Nat Med. 2026 Jul 7;32(7):2533–45. doi: 10.1038/s41591-026-04472-w (PMC13375543; doi:10.1038/s41591-026-04472-w)
Supplement: Supplementary file 2 — Reporting Summary [file 41591_2026_4472_MOESM2_ESM.pdf]

nature portfolio

Corresponding author(s): Deborah Theodore

Last updated by author(s): Apr 27, 2026

Reporting Summary

Nature Portfolio wishes to improve the reproducibility of the work that we publish. This form provides structure for consistency and transparency in reporting. For further information on Nature Portfolio policies, see our Editorial Policies and the Editorial Policy Checklist.

Statistics

For all statistical analyses, confirm that the following items are present in the figure legend, table legend, main text, or Methods section.

n/a	Confirmed	
☒	☐	The exact sample size (*n*) for each experimental group/condition, given as a discrete number and unit of measurement
☒	☐	A statement on whether measurements were taken from distinct samples or whether the same sample was measured repeatedly
☒	☐	The statistical test(s) used AND whether they are one- or two-sided<br>*Only common tests should be described solely by name; describe more complex techniques in the Methods section.*
☒	☐	A description of all covariates tested
☒	☐	A description of any assumptions or corrections, such as tests of normality and adjustment for multiple comparisons
☒	☐	A full description of the statistical parameters including central tendency (e.g. means) or other basic estimates (e.g. regression coefficient) AND variation (e.g. standard deviation) or associated estimates of uncertainty (e.g. confidence intervals)
☒	☐	For null hypothesis testing, the test statistic (e.g. *F*, *t*, *r*) with confidence intervals, effect sizes, degrees of freedom and *P* value noted<br>*Give P values as exact values whenever suitable.*
☒	☐	For Bayesian analysis, information on the choice of priors and Markov chain Monte Carlo settings
☒	☐	For hierarchical and complex designs, identification of the appropriate level for tests and full reporting of outcomes
☒	☐	Estimates of effect sizes (e.g. Cohen's *d*, Pearson's *r*), indicating how they were calculated

Our web collection on statistics for biologists contains articles on many of the points above.

Software and code

Policy information about availability of computer code

Data collection	All study data were collected by the clinical study staff using designated source documents and entered onto the appropriate electronic case report forms. Data collection forms were provided by Emmes.
Data analysis	Analyses were performed in SAS 9.4 for safety and tolerability analyses. Analyses were performed in R (v4.4.0) using the tidyverse (v2.0.0) and MonolixSuite (v2024R1, Lixoft) for population pharmacokinetic modeling. Signal data for anti-drug antibody characterization were analyzed using SoftMax Pro GxP software. No custom algorithms or unpublished software were used. All R code required to reproduce the results are available at https://github.com/HyrienLab/ABA0101Manuscript/, and will be provided to editors and reviewers during peer review. The code supporting the shiny application is available at https://github.com/FredHutch/Sim10e8imab.

For manuscripts utilizing custom algorithms or software that are central to the research but not yet described in published literature, software must be made available to editors and reviewers. We strongly encourage code deposition in a community repository (e.g. GitHub). See the Nature Portfolio guidelines for submitting code & software for further information.

Data

Policy information about availability of data

All manuscripts must include a data availability statement. This statement should provide the following information, where applicable:

- Accession codes, unique identifiers, or web links for publicly available datasets
- A description of any restrictions on data availability
- For clinical datasets or third party data, please ensure that the statement adheres to our policy

The full analyzable data set (all participant-level data collected in the study, including safety and immunogenicity data) and all versions of the protocol used in the trial will be available indefinitely through the Vivli platform at https://doi.org/10.25934/PR00012686.

Research involving human participants, their data, or biological material

Policy information about studies with human participants or human data. See also policy information about sex, gender (identity/presentation), and sexual orientation and race, ethnicity and racism.

Reporting on sex and gender	Sex and gender were determined based on self report. To ensure that volunteers assigned male and female at birth were adequately represented in the study, the study design stipulated that the trial would seek to enroll at least approximately 40% of each sex assigned at birth overall. Among the study participants, 48% were assigned female sex at birth and 52% were assigned male sex at birth. No sex- or gender-based analyses were planned, as the small number of participants overall was insufficient to enable meaningful sub analyses.
Reporting on race, ethnicity, or other socially relevant groupings	Race and ethnicity data were based on participant self-report. Half of participants identified as White (50.0%), followed by Black or African American (16.7%) and Other/Unknown/Refused to specify (16.7%), Asian (14.8%), and Multiracial (1.8%). Twenty-one participants (38.9%) reported Latino or Hispanic ethnicity.
Population characteristics	Median participant age was 27.5 years old. Nine participants were living with HIV. The remaining participants (n=45) were not living with HIV.
Recruitment	Participants were recruited through flyers posted in community spaces, within HIV clinics (providers for people living with HIV were alerted to their patients' potential eligibility; interested patients were referred to the research team), in-person recruiting (e.g., at restaurants and farmers markers), social media campaigns, and advertisements posted online. Self-selection bias was likely present, but this is not anticipated to impact results significantly.
Ethics oversight	The study was approved by the Institutional Review Board of Columbia University Irving Medical Center and, for the Orlando Immunology Clinic, the Advarra Institutional Review Board. The study was overseen by an independent Safety Monitoring Committee. All participants provided written informed consent. The Community Advisory Board of the Columbia Collaborative Clinical Trials Unit provided input regarding community engagement and trial conduct. Participants were compensated for their time.

Note that full information on the approval of the study protocol must also be provided in the manuscript.

Field-specific reporting

Please select the one below that is the best fit for your research. If you are not sure, read the appropriate sections before making your selection.

☒ Life sciences ☐ Behavioural & social sciences ☐ Ecological, evolutionary & environmental sciences

For a reference copy of the document with all sections, see nature.com/documents/nr-reporting-summary-flat.pdf

Life sciences study design

All studies must disclose on these points even when the disclosure is negative.

Sample size	The sample size per group was determined based on the probabilities of observing adverse events. The number of participants per group who received an administration of 10E8.4/iMab varied from 3 to 6, depending on group assignment. Assessment of injection site reaction dose limiting toxicity in Arm 4 (subcutaneous dosing) was based on a group size of 9 (6 active and 3 placebos). The power analysis therefore considered groups of size n = 3, n = 6, and n = 9 to understand the probability of observing AEs when the true event rate ranged from 1 to 30 percent. The sample size of 54 participants across study arms allowed a descriptive statistical analysis of study data.
Data exclusions	This was a modified intent-to-treat analysis in that individuals who received a group assignment but did not receive 10E8.4/iMab or placebo did not contribute data and hence were excluded from subsequent analysis.
Replication	This section does not directly apply to our study, which is a clinical trial involving unique participants who cannot be replicated.
Randomization	Participants in Arm 4 were randomized in a 2:1 ratio (using 3 blocks of size 3) to receive either 10E8.4/iMab or placebo. Randomization was conducted by Emmes using SAS 9.4.

Arm 1 Group A (0.3 mg/kg IV, PWOH; n=3) participants enrolled first. Arm 1 Group B (1 mg/kg SC, PWOH; n=3) enrolled next. Arm 1 Group C (1 mg/kg IV, PWOH; n=3) enrolled next. Thereafter, participants in Arm 4 Group J (2.5mg/kg SC or placebo, PWOH; n=9) and Arm 2 Group D (3 mg/kg IV, PWOH; n=6) enrolled contemporaneously; if a participant was eligible for both groups and a group assignment was open for both, the participant could choose whether to enter Arm 4 Group J (and thereafter undergo randomization to 10E8.4/iMab or placebo) or Arm 2 Group D. Next, participants in Arm 4 Group K (10 mg/kg SC or placebo, PWOH; n=9) and Arm 2 Group E (10 mg/kg IV, PWOH; n=6) enrolled contemporaneously; similarly, if a participant was eligible for both groups and a group assignment was open for both, the participant could choose whether to enter Arm 4 Group K (and thereafter undergo randomization to 10E8.4/iMab or placebo) or Arm 2 Group E. Next, participants in Arm 2 Group F (30 mg/kg IV, PWOH; n=6) and Arm 3 Group H (10 mg/kg IV, PWH with active viremia; n=3) enrolled contemporaneously, with group assignment determined by HIV status. Finally, participants in Arm 3a Group I (30 mg/kg IV, PWH with viral suppression; n=6) enrolled.

Blinding

Blinding was not applicable for Arms 1-3, as these arms did not include placebo recipients. In Arm 4 (in which participants were randomized to receive 10E8.4/iMab or placebo), participants and investigators (except for the unblinded site pharmacist) were blinded to treatment assignment (i.e., 10E8.4/iMab versus placebo) until day 84; all data collection in Arm 4 was carried out under the blind until day 84. The primary purpose of blinding was to assess safety and tolerability of the study product when received subcutaneously, in comparison to subcutaneous placebo. In order to evaluate this, it was not possible to maintain the blind during data analysis, as safety and tolerability were analyzed based on treatment assignment. Additionally, antiviral and PK/PD measurements were not performed for placebo recipients, so these analyses were performed unblinded to treatment assignment. ADA measurements were conducted while investigators were blinded to Arm 4 treatment assignment, but final manuscript figures were created when investigators were aware of study assignment in order to facilitate presentation of the relevant results.

Reporting for specific materials, systems and methods

We require information from authors about some types of materials, experimental systems and methods used in many studies. Here, indicate whether each material, system or method listed is relevant to your study. If you are not sure if a list item applies to your research, read the appropriate section before selecting a response.

Materials & experimental systems

n/a	Involved in the study	
☐	☒	Antibodies
☒	☐	Eukaryotic cell lines
☒	☐	Palaeontology and archaeology
☒	☐	Animals and other organisms
☐	☒	Clinical data
☒	☐	Dual use research of concern
☒	☐	Plants

Methods

n/a	Involved in the study	
☒	☐	ChIP-seq
☐	☒	Flow cytometry
☒	☐	MRI-based neuroimaging

Antibodies

Antibodies used

The investigational product was a bispecific antibody (10E8.4/iMab) manufactured by WuXi AppTec Biopharmaceuticals Co., Ltd, IND Number: 141672. The IND Sponsor was Dr. David D. Ho, Aaron Diamond AIDS Research Center at Columbia University Irving Medical Center. Positive controls for functional ADA assays included mouse anti-idiotype mAbs specific for 10E8.4 or iMab.

10E8.4/iMab monoclonal (bispecific) antibody drug product (Source: WuXi Biologics) - Lot # 201801003. For the 10E8.4/iMab used in the ADA binding assay, the final concentration of each drug conjugate is 0.667 ug/ml and serum is diluted 1:12.
Anti-10E8.4 (Source: Syngene International Limited) - Lot # PRB010089, clone name: M30E12B6
Anti-iMab (Source: TaiMed Biologics) - Lot # 020-JZ-6-168
iMab, 10E8.4, and 10-1074/3BNC117 CrossMab (Source: ADARC)

Validation

10E8.4/iMab expression, production and purification is described in the protocol as follows: Plasmid DNA encoding the 10E8.4/iMab antibody were stably transfected into CHO K1 cells. The preparation of the CHO K1 host cell was performed at the Shanghai site of WuXi Biologics.
Cells were screened after stable transfection to down-select a single clone expressing high titers of 10E8.4/iMab with satisfactory product quality attributes.
The Master Cell Bank (MCB), WBP2063B-30-M01-009-MCB was generated from a parental cell bank at the GMP cell banking facility at the Shanghai site of WuXi Biologics. The MCB vials are currently stored in vapor phase LN2 storage units at the Shanghai site of WuXi Biologics.
Cell banks were characterized based on the Guidance for Industry entitled “Points to Consider in the Manufacture and Testing of Monoclonal Antibody Products for Human Use” and ICH Q5A and Q5D. Testing was performed in compliance with US FDA GLP regulations 21 CFR 58 and cGMP regulations 21 CFR Parts 210 and 211 as specified in the characterization test reports.
The cell viability, cell productivity, and metabolic and pH profile data obtained during the GMP drug substance manufacture run, and the product quality attributes of 10E8.4/iMab purified from this GMP drug substance manufacture run, were comparable to those of previous 15 L bioreactor development runs and 200 L bioreactor engineering run, suggesting that the 10E8.4/iMab cell culture process and purification process are repeatable and robust.

Clinical data

Policy information about clinical studies

All manuscripts should comply with the ICMJE guidelines for publication of clinical research and a completed CONSORT checklist must be included with all submissions.

Clinical trial registration	This study was registered at ClinicalTrials.gov, number NCT03875209, on March 14, 2019.
Study protocol	The full study protocol is included as an attachment.
Data collection	The study was conducted at two centers in the US (Columbia University Irving Medical Center [CUIMC] in New York and Orlando Immunology Center [OIC] in Florida) between March 21, 2019 and October 1, 2021, with all recruitment and data collection taking place during that time period.
Outcomes	Outcomes were predefined in the study protocol. The primary outcome was the rate of signs, symptoms and laboratory abnormalities, in addition to local and systemic solicited adverse events (AEs), within 2 weeks of 10E8.4/iMab administration in all study arms/groups. Secondary outcomes were the PK profile of 10E8.4/iMab (elimination half-life [t1/2], clearance [CL/F], volume of distribution [Vz/F], area under the curve [AUC] and decay curve in all study arms/groups); the decline in plasma HIV-1 RNA level by standard clinical assay after 10E8.4/iMab infusion in PWH with viremia; the frequency and levels of induced anti-10E8.4/iMab antibodies in all study groups; the rate of signs, symptoms and laboratory abnormalities that occurred during study follow up after 10E8.4/iMab infusion/injection in all study groups; and the absolute and relative CD4+ and CD8+ T cell counts after 10E8.4/iMab infusion.

Plants

Seed stocks	*Report on the source of all seed stocks or other plant material used. If applicable, state the seed stock centre and catalogue number. If plant specimens were collected from the field, describe the collection location, date and sampling procedures.*
Novel plant genotypes	*Describe the methods by which all novel plant genotypes were produced. This includes those generated by transgenic approaches, gene editing, chemical/radiation-based mutagenesis and hybridization. For transgenic lines, describe the transformation method, the number of independent lines analyzed and the generation upon which experiments were performed. For gene-edited lines, describe the editor used, the endogenous sequence targeted for editing, the targeting guide RNA sequence (if applicable) and how the editor was applied.*
Authentication	*Describe any authentication procedures for each seed stock used or novel genotype generated. Describe any experiments used to assess the effect of a mutation and, where applicable, how potential secondary effects (e.g. second site T-DNA insertions, mosiacism, off-target gene editing) were examined.*

Flow Cytometry

Plots

Confirm that:

- [x] The axis labels state the marker and fluorochrome used (e.g. CD4-FITC).
- [x] The axis scales are clearly visible. Include numbers along axes only for bottom left plot of group (a 'group' is an analysis of identical markers).
- [x] All plots are contour plots with outliers or pseudocolor plots.
- [x] A numerical value for number of cells or percentage (with statistics) is provided.

Methodology

Sample preparation	Fresh whole blood was incubated with 10E8.4/iMab and stained with a multicolor antibody panel followed by RBC lysis and fixation before acquisition.
Instrument	Instrument: BD LSR II SORP Flow Cytometer
Software	FACSDiva Version 6.2 and later
Cell population abundance	50,000 lymphocytes
Gating strategy	The gating strategy is defined in the attached CD4RO assay protocol.

- [x] Tick this box to confirm that a figure exemplifying the gating strategy is provided in the Supplementary Information.
